# The Risk of Muscular Events Among New Users of Hydrophilic and Lipophilic Statins: an Observational Cohort Study

**DOI:** 10.1007/s11606-021-06651-6

**Published:** 2021-03-09

**Authors:** Alexandra M. Mueller, Evangelia Liakoni, Cornelia Schneider, Theresa Burkard, Susan S. Jick, Stephan Krähenbühl, Christoph R. Meier, Julia Spoendlin

**Affiliations:** 1 Basel Pharmacoepidemiology Unit, Division of Clinical Pharmacy and Epidemiology, Department of Pharmaceutical Sciences, University of Basel, Basel, Switzerland; 2 Hospital Pharmacy, University Hospital Basel, Basel, Switzerland; 3 Clinical Pharmacology and Toxicology, Department of General Internal Medicine, Inselspital, Bern University Hospital, University of Bern, Bern, Switzerland; 4 Boston Collaborative Drug Surveillance Program, Lexington, MA USA; 5 Boston University School of Public Health, Boston, MA USA; 6 Division of Clinical Pharmacology and Toxicology, University Hospital Basel, Basel, Switzerland; 7 Department of Biomedicine, University of Basel, Basel, Switzerland

**Keywords:** statin, myopathy, myalgia, adverse events, CPRD

## Abstract

**Background:**

Statins are effective lipid-lowering drugs for the prevention of cardiovascular disease, but muscular adverse events can limit their use. Hydrophilic statins (pravastatin, rosuvastatin) may cause less muscular events than lipophilic statins (e.g. simvastatin, atorvastatin) due to lower passive diffusion into muscle cells.

**Objective:**

To compare the risk of muscular events between statins at comparable lipid-lowering doses and to evaluate if hydrophilic statins are associated with a lower muscular risk than lipophilic statins.

**Design/Setting:**

Propensity score-matched cohort study using data from the United Kingdom-based Clinical Practice Research Datalink (CPRD) GOLD.

**Patients:**

New statin users. Cohort 1: pravastatin 20-40 mg (hydrophilic) vs simvastatin 10-20 mg (lipophilic), cohort 2: rosuvastatin 5-40 mg (hydrophilic) vs atorvastatin 10-80 mg (lipophilic), and cohort 3: simvastatin 40-80 mg vs atorvastatin 10-20 mg.

**Main Measures:**

The outcome was a first record of a muscular event (myopathy, myalgia, myositis, rhabdomyolysis) during a maximum follow-up of 1 year.

**Key Results:**

The propensity score-matched cohorts consisted of 1) 9,703, 2) 7,032, and 3) 37,743 pairs of statin users. Comparing the risk of muscular events between low-intensity pravastatin vs low-intensity simvastatin yielded a HR of 0.86 (95% CI 0.64-1.16). In the comparison of moderate- to high-intensity rosuvastatin vs equivalent doses of atorvastatin, we observed a HR of 1.17 (95% CI 0.88-1.56). Moderate- to high-intensity simvastatin was associated with a HR of 1.33 (95% CI 1.16-1.53), when compared with atorvastatin at equivalent doses.

**Limitations:**

We could not conduct other pairwise comparisons of statins due to small sample size. In the absence of a uniform definition on the comparability of statin doses, the applied dose ratios may not fully match with all literature sources.

**Conclusions:**

Our results do not suggest a systematically lower risk of muscular events for hydrophilic statins when compared to lipophilic statins at comparable lipid-lowering doses.

**Supplementary Information:**

The online version contains supplementary material available at 10.1007/s11606-021-06651-6.

## INTRODUCTION

Statins are effective lipid-lowering drugs for the primary and secondary prevention of ischemic cardiovascular events.^1, 2^ Although generally well tolerated, statins may cause myalgia, and less frequently myositis or rhabdomyolysis.^3^ According to randomized controlled trials (RCTs), 1.5–5.0% of patients experience adverse muscular symptoms during statin treatment.^4^ However, observational studies suggested that in routine care up to 10–15% of statin users experience such adverse events.^2, 4^

The myotoxicity of statins appears to be dose-dependent and may differ across individual statins,^5, 6^ as suggested by the market withdrawal of cerivastatin due to its pronounced risk of rhabdomyolysis.^7^ It has been hypothesized that hydrophilic/water-soluble statins (i.e., rosuvastatin and pravastatin) are less likely to cause muscular side effects than lipophilic/fat-soluble statins (e.g., simvastatin or atorvastatin) due to lower passive diffusion into muscle cells.^8, 9^ This hypothesis is supported by in vitro data showing a higher cytotoxicity on C2C12 myotubes for lipophilic statins than hydrophilic statins.^10^

Results from RCTs, however, suggest a similar muscular risk for hydrophilic and lipophilic statins at comparable lipid-lowering doses, but the absolute numbers of events in these trials were low.^11, 12^ More comprehensive and robust data on the comparative muscular risks of statins at equivalent doses are needed to improve our understanding of the role of statin choice for the risk of such adverse events.

We aimed to compare the risk of muscular events between statins at comparable lipid-lowering doses, and to evaluate if hydrophilic statins are associated with a systematically lower risk of muscular events than lipophilic statins.

## METHODS

### Study Design and Data Resource

We conducted an observational cohort study using data from the United Kingdom-based Clinical Practice Research Datalink (CPRD) GOLD. CPRD GOLD is a primary care database^13^ that contains anonymized longitudinal electronic medical records on more than 15 million patients.^14, 15^ The data held in the CPRD GOLD are collected in general practices as part of routine care.^15^ Data on demographics, medical diagnoses or symptoms (using “Read codes”), or lifestyle factors are available. CPRD GOLD contains comprehensive and detailed information on drug prescriptions, including product name, dose, number of tablets, and prescription date.^15^

The study protocol was approved by the Independent Scientific Advisory Committee for Medicines and Healthcare Products Regulatory Agency database research (protocol no. 19_052) and has been made available to the journal editors.

### Study Population

We identified all patients in CPRD GOLD aged 40 to 80 years with a first statin prescription between January 2000 and December 2017. Of these, we selected all patients with a first prescription for hydrophilic pravastatin 20–40 mg, hydrophilic rosuvastatin 5–40 mg, lipophilic atorvastatin 10–80 mg, or lipophilic simvastatin 10–80 mg who did not have any of the following exclusion criteria at the cohort entry date (CED), defined as date of the first statin prescription: (i) < 3 years of medical records in CPRD GOLD; (ii) prior record of rhabdomyolysis, myositis, or myopathy (except myalgia, muscle ache, or muscle pain); (iii) prior diagnosis of a primary muscle disorder, myoneural disorder, or a disorder associated with muscle pain (e.g., polymyalgia rheumatica, fibromyalgia); and/or (iv) any diagnosis of cancer (except non-melanoma skin cancer), alcoholism or other substance abuse, or HIV.

To minimize heterogeneity in patient frailty, we categorized patients into two groups of (i) primary or (ii) secondary prevention of cardiovascular disease. Those with a diagnosis of myocardial infarction or ischemic stroke before the CED were classified as secondary prevention. All others were classified as primary prevention. Within each of the two groups, we established 3 cohorts (6 cohorts in total) to conduct pairwise comparisons of statins at comparable lipid-lowering doses: cohort 1—new users of pravastatin 20–40 mg vs simvastatin 10–20 mg (low-intensity statin therapy); cohort 2—new users of rosuvastatin 5–40 mg vs atorvastatin 10–80 mg; and cohort 3—new users of simvastatin 40–80 mg vs atorvastatin 10–20 mg (moderate- to high-intensity statin therapy). We could not conduct other pairwise comparisons due to small sample size. We based comparability of doses on national guidelines in the UK.^1^ Because rosuvastatin was only launched in the UK in March 2003,^16^ the CED of atorvastatin users in cohort 2 had to be at or after that date. New users of atorvastatin 10–20 mg may have been included in cohorts 2 and 3.

### Statin Exposure Measurement and Follow-up

We followed patients in an “as treated” approach from the day after CED for a maximum of 1 year or until the occurrence of an outcome (muscular event). We censored patients 90 days after statin discontinuation (no prescription re-fill for the study drug within 90 days after the estimated end date of statin supply^17^), on the day of treatment switch (prescription for a type of statin other than the study drug), death, disenrollment from CPRD GOLD, the day of a recorded exclusion criterion, a recording of statin intolerance (if not classified as outcome, see below), or at the end of the study period (December 31, 2017). We introduced the 90-day grace period for censoring after statin discontinuation to account for potentially delayed recording of outcomes. We calculated the supply of a statin prescription based on the prescribed number of tablets and on an assumed regimen of 1 tablet per day.

### Study Outcome

The outcome of interest was a muscular event, defined as a recorded Read code for (i) myopathy (proximal, drug-induced, or toxic), myalgia (including “muscle pain” or “muscle ache”), myositis, rhabdomyolysis, or unspecified muscle disorder, or (ii) statin intolerance, if followed by a Read code listed under (i) within 90 days (*N* = 32). Read codes are listed in Appendix Table 1.

### Covariates

We assessed 42 baseline covariates^7, 18–21^ before the CED including demographics, lifestyle factors, body mass index, comorbidities, comedication, health care utilization, and the initially prescribed statin dose (Appendix Table 2).

### Statistical Analysis

Within each of the 6 cohorts, we performed propensity score (PS) matching, which is an established method to control for confounding by balancing assessed baseline covariates between comparison groups.^22, 23^ Assessed baseline covariates were potential confounders or predictors of the risk of muscular events.^22^ For each patient, we calculated a PS, i.e., the predicted probability of receiving the statin of interest over the comparator statin based on all assessed baseline covariates, using multivariable logistic regression (dependent variable: treatment group; predictor variables: assessed baseline covariates). To account for potential bias due to changes in statin prescribing practice over time,^1, 24, 25^ we calculated calendar time-specific PS, i.e., performed PS calculation separately within 2-year time intervals, each including the patients with a CED during that time period.^26^ We matched users of a statin of interest 1:1 to users of a comparator statin with a comparable PS within the 2-year time interval, applying a greedy 5-to-1 digit matching algorithm. This algorithm initially matches on 5 digits of the PS and, in each iteration, on a further reduced number of digits to match the previously unmatched statin users. Statin users who could not be matched were excluded. It has been shown that treatment groups with the same distribution of propensity scores have the same distribution of all assessed baseline covariates.^27^ Covariate balance before and after PS matching was assessed using absolute standardized differences (ASD). We defined covariate balance as an ASD <10%.^28^ We plotted Kaplan-Meier curves in the matched cohorts and performed Cox proportional hazard analyses to calculate hazard ratios (HRs) with 95% confidence intervals (CIs). As part of the primary analysis, we calculated time-specific HRs for the follow-up periods of 1 to 30 days, 31 to 90 days, 91 to 180 days, and 181 to 365 days in the primary prevention cohorts.

We performed subgroup analyses by sex, age, and initial daily statin dose and conducted sensitivity analyses restricted to patients with (i) no muscle complaints before the CED and (ii) no use of CYP3A4 inhibiting drugs. CYP3A4-mediated interactions with simvastatin and atorvastatin have been described as a clinically relevant cause of muscular adverse events.^7, 29, 30^ In further analyses, we (i) additionally censored for change in statin dose and (ii) applied a broader outcome definition including all “statin intolerance” records. Finally, we repeated our analyses as multivariable regression analyses. Appendix Table 3 provides further information on the additionally performed analyses. We conducted the majority of additional analyses only in the primary prevention cohorts due to the small sample size of the secondary prevention cohorts. All analyses were conducted using SAS version 9.4 (SAS Institute, Cary, NC, USA).

## RESULTS

### Cohort Characteristics

We identified 553,178 eligible statin initiators, of which we grouped 469,860 (84.9%) patients into the primary prevention group and 83,318 (15.1%) patients into the secondary prevention group (Appendix Figure 1). In the primary prevention group, we obtained the following PS-matched cohorts: (i) 9,703 pairs of pravastatin 20–40 mg vs simvastatin 10–20 mg, (ii) 7,032 pairs of rosuvastatin 5–40 mg vs atorvastatin 10–80 mg, and (iii) 37,743 pairs of simvastatin 40–80 mg vs atorvastatin 10–20 mg. The corresponding cohorts in the secondary prevention group included (iv) 4,121, (v) 836, and (vi) 6,716 pairs.

In all 6 cohorts, covariate balance was achieved after PS matching (Table 1, Appendix Tables 4–7; censoring reasons after PS matching in Table 2 and Appendix Table 8). Kaplan Meier plots for the primary prevention cohorts are displayed in Appendix Figure 2. Sample size, covariate distribution, and censoring reasons for the cohorts before PS matching are shown in the Appendix Tables 4–10.

**Table 1:** Baseline Covariates by Treatment Group in the Primary Prevention Cohorts of Pravastatin vs Simvastatin and Rosuvastatin vs Atorvastatin After Propensity Score Matching (Simvastatin vs Atorvastatin in Appendix Table 4)

Covariate	Low-intensity statin therapy			Moderate- to high-intensity statin therapy		
	Pravastatin *N* = 9,703	Simvastatin *N* = 9,703	ASD (%)	Rosuvastatin *N* = 7,032	Atorvastatin *N* = 7,032	ASD (%)
Age (years), mean (SD)	63.1 (9.8)	63.0 (9.8)	1.1	61.0 (9.9)	61.1 (9.9)	−1.0
Male, *n* (%)	4,887 (50.4)	4,960 (51.1)	−1.5	3,411 (48.5)	3,364 (47.8)	1.3
Current smoker*, *n* (%)	1,950 (20.1)	2,009 (20.7)	−1.5	1,376 (19.6)	1,321 (18.8)	2.0
>14 alcohol units/week*, *n* (%)	809 (8.3)	824 (8.5)	−0.6	709 (10.1)	698 (9.9)	0.5
Obesity*, *n* (%)	2,531 (26.1)	2,547 (26.2)	−0.4	2,104 (29.9)	2,109 (30.0)	−0.2
Comorbidities, *n* (%)—at any time before the cohort entry date, if not specified otherwise						
Hyperlipidemia	4,912 (50.6)	4,867 (50.2)	0.9	4,877 (69.4)	4,916 (69.9)	−1.2
Diabetes mellitus	2,097 (21.6)	2,165 (22.3)	−1.7	1,505 (21.4)	1,497 (21.3)	0.3
Hypertension	4,750 (49.0)	4,754 (49.0)	−0.1	3,614 (51.4)	3,663 (52.1)	−1.4
Heart failure	400 (4.1)	375 (3.9)	1.3	116 (1.6)	124 (1.8)	−0.9
Atrial fibrillation	790 (8.1)	716 (7.4)	2.9	216 (3.1)	219 (3.1)	−0.2
Ischemic heart disease	2,629 (27.1)	2,619 (27.0)	0.2	698 (9.9)	713 (10.1)	−0.7
Peripheral arterial disease	492 (5.1)	464 (4.8)	1.3	148 (2.1)	132 (1.9)	1.6
Hemorrhagic stroke	82 (0.8)	99 (1.0)	−1.8	30 (0.4)	29 (0.4)	0.2
Chronic kidney disease	575 (5.9)	535 (5.5)	1.8	482 (6.9)	487 (6.9)	−0.3
Severe liver impairment	27 (0.3)	34 (0.4)	−1.3	X	5 (0.1)	X
Hypothyroidism	658 (6.8)	641 (6.6)	0.7	521 (7.4)	505 (7.2)	0.9
Hyperthyroidism	161 (1.7)	160 (1.6)	0.1	104 (1.5)	103 (1.5)	0.1
Rheumatoid arthritis	166 (1.7)	163 (1.7)	0.2	97 (1.4)	107 (1.5)	−1.2
Osteoarthritis	1,862 (19.2)	1,812 (18.7)	1.3	1,242 (17.7)	1,272 (18.1)	−1.1
Pre-existing muscle complaints	842 (8.7)	830 (8.6)	0.4	574 (8.2)	571 (8.1)	0.2
Musculoskeletal injuries	2,821 (29.1)	2,843 (29.3)	−0.5	2,139 (30.4)	2,139 (30.4)	0.0
COPD	439 (4.5)	446 (4.6)	−0.3	228 (3.2)	238 (3.4)	−0.8
Macular degeneration	84 (0.9)	82 (0.8)	0.2	37 (0.5)	41 (0.6)	−0.8
Falls^†^	265 (2.7)	256 (2.6)	0.6	175 (2.5)	189 (2.7)	−1.3
Pressure ulcer^†^	54 (0.6)	60 (0.6)	−0.8	26 (0.4)	19 (0.3)	1.8
Incontinence^†^	70 (0.7)	66 (0.7)	0.5	53 (0.8)	59 (0.8)	−1.0
Peripheral venous thrombosis^†^	155 (1.6)	169 (1.7)	−1.1	98 (1.4)	100 (1.4)	−0.2
Pneumonia^†^	66 (0.7)	71 (0.7)	−0.6	27 (0.4)	33 (0.5)	−1.3
Dysphagia^†^	65 (0.7)	56 (0.6)	1.2	30 (0.4)	34 (0.5)	−0.8
Anemia^†^	207 (2.1)	230 (2.4)	−1.6	94 (1.3)	91 (1.3)	0.4
Comedication, *n* (%)—in the 180 days before the cohort entry date						
Fibrates	131 (1.4)	138 (1.4)	−0.6	174 (2.5)	175 (2.5)	−0.1
Amiodarone	185 (1.9)	157 (1.6)	2.2	21 (0.3)	24 (0.3)	−0.8
Systemic corticosteroids	380 (3.9)	400 (4.1)	−1.0	215 (3.1)	223 (3.2)	−0.7
Antipsychotics	41 (0.4)	39 (0.4)	0.3	44 (0.6)	41 (0.6)	0.6
H_2_-receptor antagonists	539 (5.6)	531 (5.5)	0.4	196 (2.8)	204 (2.9)	−0.7
Benzodiazepines	842 (8.7)	810 (8.3)	1.2	527 (7.5)	520 (7.4)	0.4
Number of cardiovascular drug classes						
0	1,760 (18.1)	1,788 (18.4)	−0.7	2,240 (31.9)	2,197 (31.2)	1.3
1 to 3	5,807 (59.8)	5,835 (60.1)	−0.6	4,063 (57.8)	4,059 (57.7)	0.1
4 to 10	2,136 (22.0)	2,080 (21.4)	1.4	729 (10.4)	776 (11.0)	−2.2
Number of general practitioner visits^‡^, mean (SD)	20.6 (13.4)	20.6 (13.1)	−0.1	19.0 (12.2)	19.0 (12.6)	0.3
Hospitalization^†^, *n* (%)	2,743 (28.3)	2,787 (28.7)	−1.0	1,727 (24.6)	1,717 (24.4)	0.3
Daily statin dose (mg), *n* (%)						
20 (P), 10 (S)	4,405 (45.4)	4,453 (45.9)	−1.0	NA	NA	
40 (P), 20 (S)	5,298 (54.6)	5,250 (54.1)	1.0	NA	NA	
40 (S), 5 (R), 10 (A)	NA	NA		695 (9.9)	714 (10.2)	−0.9
80 (S), 10 (R), 20 (A)	NA	NA		6,088 (86.6)	6,069 (86.3)	0.8
20 (R), 40 (A)	NA	NA		226 (3.2)	228 (3.2)	−0.2
40 (R), 80 (A)	NA	NA		23 (0.3)	21 (0.3)	0.5
Cohort entry date, *n* (%)						
2000–2001	2,912 (30.0)	2,912 (30.0)	0.0	NA	NA	
2002–2003	4,202 (43.3)	4,202 (43.3)	0.0	1,410 (20.1)	1,410 (20.1)	0.0
2004–2005	1,408 (14.5)	1,408 (14.5)	0.0	3,132 (44.5)	3,132 (44.5)	0.0
2006–2007	321 (3.3)	321 (3.3)	0.0	1,263 (18.0)	1,263 (18.0)	0.0
2008–2009	214 (2.2)	214 (2.2)	0.0	556 (7.9)	556 (7.9)	0.0
2010–2011	246 (2.5)	246 (2.5)	0.0	338 (4.8)	338 (4.8)	0.0
2012–2013	284 (2.9)	284 (2.9)	0.0	144 (2.0)	144 (2.0)	0.0
2014–2015	88 (0.9)	88 (0.9)	0.0	110 (1.6)	110 (1.6)	0.0
2016–2017	28 (0.3)	28 (0.3)	0.0	79 (1.1)	79 (1.1)	0.0

*ASD* absolute standardized difference, *SD* standard deviation, *X* cell contains <5 patients (not shown owing to ethics regulations to preserve confidentiality), *COPD* chronic obstructive pulmonary disease, *P* pravastatin, *S* simvastatin, *R* rosuvastatin, *A* atorvastatin, *NA* not applicable

*Last record before the cohort entry date

†Assessed in the 3 years before the cohort entry date

‡Assessed in the 1 year before the cohort entry date

**Table 2:** Censoring Reasons and Duration of Follow-up for the Primary Prevention Cohorts After Propensity Score Matching

**Low-intensity statin therapy**		
	**Pravastatin 20–40 mg**	**Simvastatin 10–20 mg**
	***N*= 9,703**	***N*= 9,703**
Censoring reasons, *n* (%)		
Muscular event (outcome)	82 (0.8)	98 (1.0)
Recording of statin intolerance	17 (0.2)	47 (0.5)
Treatment switch	1,287 (13.3)	716 (7.4)
Discontinuation of statin treatment	1,758 (18.1)	1,802 (18.6)
Death	40 (0.4)	26 (0.3)
Recording of an exclusion criterion	198 (2.0)	175 (1.8)
Myocardial infarction or ischemic stroke	283 (2.9)	206 (2.1)
End of study enrollment	6,038 (62.2)	6,633 (68.4)
Duration of follow-up		
Mean number of days (standard deviation)	285.0 (112.5)	295.4 (109.1)
Median number of days (interquartile range)	365 (183–365)	365 (212–365)
**Moderate- to high-intensity statin therapy**		
	**Rosuvastatin 5**–**40 mg**	**Atorvastatin 10**–**80 mg**
	***N*= 7,032**	***N*= 7,032**
Censoring reasons, *n* (%)		
Muscular event (outcome)	100 (1.4)	86 (1.2)
Recording of statin intolerance	20 (0.3)	22 (0.3)
Treatment switch	678 (9.6)	661 (9.4)
Discontinuation of statin treatment	1,408 (20.0)	1,378 (19.6)
Death	8 (0.1)	15 (0.2)
Recording of an exclusion criterion	126 (1.8)	135 (1.9)
Myocardial infarction or ischemic stroke	59 (0.8)	81 (1.2)
End of study enrollment	4,633 (65.9)	4,654 (66.2)
Duration of follow-up		
Mean number of days (standard deviation)	292.1 (110.5)	293.7 (108.3)
Median number of days (interquartile range)	365 (205–365)	365 (208–365)
	**Simvastatin 40**–**80 mg**	**Atorvastatin 10**–**20 mg**
	***N*= 37,743**	***N*= 37,743**
Censoring reasons, *n* (%)		
Muscular event (outcome)	483 (1.3)	368 (1.0)
Recording of statin intolerance	214 (0.6)	166 (0.4)
Treatment switch	3,529 (9.4)	3,156 (8.4)
Discontinuation of statin treatment	7,279 (19.3)	7,526 (19.9)
Death	110 (0.3)	91 (0.2)
Recording of an exclusion criterion	686 (1.8)	651 (1.7)
Myocardial infarction or ischemic stroke	713 (1.9)	467 (1.2)
End of study enrollment	24,729 (65.5)	25,318 (67.1)
Duration of follow-up		
Mean number of days (standard deviation)	282.6 (115.4)	287.3 (110.8)
Median number of days (interquartile range)	365 (176–365)	365 (188–365)

## Comparative Safety

### Primary Prevention Group

#### Low-Intensity Statin Therapy Pravastatin (hydrophilic) vs simvastatin (lipophilic)

After PS matching, the incidence rate (IR) of muscular events was 10.8 per 1000 person-years (PYs) in pravastatin users and 12.5 per 1000 PYs in simvastatin users (Table 3). The overall HR for the risk of muscular events was 0.86 (95% CI 0.64–1.16) for pravastatin use compared with simvastatin use, which was driven by low HRs between days 1–30 (HR 0.60, 95% CI 0.26–1.37) and days 31–90 (HR 0.60, 95% CI 0.32–1.11) of follow-up. After day 90, HRs attenuated to a null result (Table 4 and Appendix Figure 3). Results were not meaningfully different within subgroups by age and statin dose. The HR was higher in women (0.99, 95% CI 0.67–1.47) than men (HR 0.73, 95% CI 0.47–1.14). The overall result did not change materially after restriction to statin users without concomitant use of CYP3A4 inhibitors, restriction to patients with no muscle complaints before the CED, and additional censoring for change in statin dose. When we applied the broader outcome definition including all records of “statin intolerance,” the HR decreased to 0.70 (95% CI 0.54–0.91) (Table 5). Findings from the multivariable regression analyses were similar to those obtained in the PS-matched cohort (Appendix Table 11).

**Table 3:** Incidence Rates of the Muscular Events in the Primary Prevention Cohorts Before and After Propensity Score Matching

	Number (%) of muscular events		Total person-years of follow-up		Incidence rate per 1,000 person-years	
	Exposed	Comparator	Exposed	Comparator	Exposed	Comparator
Low-intensity statin therapy						
Pravastatin vs simvastatin (ref)						
Crude	82 (0.8)	2,205 (1.2)	7,584	143,220	10.8	15.4
PS-matched	82 (0.8)	98 (1.0)	7,577	7,852	10.8	12.5
Moderate- to high-intensity statin therapy						
Rosuvastatin vs atorvastatin (ref)						
Crude	101 (1.4)	854 (1.0)	5,648	64,369	17.9	13.3
PS-matched	100 (1.4)	86 (1.2)	5,628	5,658	17.8	15.2
Simvastatin vs atorvastatin (ref)						
Crude	2,456 (1.5)	957 (0.9)	124,546	78,697	19.7	12.2
PS-matched	483 (1.3)	368 (1.0)	29,222	29,708	16.5	12.4

*Ref* reference, *PS* propensity score

**Table 4:** Hazard Ratios for Muscular Events in the Primary Prevention Cohorts Before and After Propensity Score Matching

	Hazard ratio (95% CI)	
	Crude	PS-matched
Low-intensity statin therapy		
Pravastatin vs simvastatin (ref)		
Overall	0.70 (0.56–0.87)	0.86 (0.64–1.16)
Time-specific* (days of follow-up)		
1–30	0.41 (0.21–0.80)	0.60 (0.26–1.37)
31–90	0.51 (0.31–0.83)	0.60 (0.32–1.11)
91–180	0.74 (0.48–1.13)	0.97 (0.54–1.74)
181–365	1.02 (0.73–1.44)	1.13 (0.70–1.82)
Moderate- to high-intensity statin therapy		
Rosuvastatin vs atorvastatin (ref)		
Overall	1.36 (1.11–1.68)	1.17 (0.88–1.56)
Time-specific* (days of follow-up)		
1–30	1.44 (0.84–2.46)	1.15 (0.55–2.42)
31–90	1.56 (1.07–2.28)	1.43 (0.82–2.50)
91–180	1.17 (0.75–1.81)	1.24 (0.66–2.31)
181–365	1.33 (0.93–1.90)	0.97 (0.60–1.57)
Simvastatin vs atorvastatin (ref)		
Overall	1.62 (1.50–1.75)	1.33 (1.16–1.53)
Time-specific* (days of follow-up)		
1–30	1.86 (1.55–2.24)	1.91 (1.29–2.81)
31–90	1.65 (1.43–1.91)	1.46 (1.13–1.88)
91–180	1.57 (1.36–1.82)	1.31 (1.00–1.71)
181–365	1.51 (1.32–1.73)	1.09 (0.86–1.38)

*CI* confidence interval, *PS* propensity score, *Ref* reference

*Patients whose follow-up ended before the time window of interest were excluded from the respective analysis. We censored patients on the day of the end of the time window of interest in any given analysis

**Table 5:** Hazard Ratios for Subgroup, Sensitivity, and Additional Analyses for Muscular Events in the Primary Prevention Cohorts After Propensity Score Matching

	Number of events		Total person-years of follow-up		HR (95% CI)
	Exposed	Comparator	Exposed	Comparator	
Low-intensity statin therapy					
Pravastatin vs simvastatin (ref)					
Subgroup analyses					
Male	33	46	3,860	3,938	0.73 (0.47–1.14)
Female	49	51	3,711	3,860	0.99 (0.67–1.47)
40–64 years	39	58	3,903	4,028	0.69 (0.46–1.04)
≥65 years	43	54	3,665	3,743	0.81 (0.54–1.21)
20 vs 10 mg	35	49	3,458	3,562	0.73 (0.47–1.13)
40 vs 20 mg	47	59	4,110	4,258	0.82 (0.56–1.21)
Sensitivity analyses					
No muscle complaints before CED	71	98	6,932	7,120	0.74 (0.55–1.01)
No use of CYP3A4 inhibiting drugs*	57	69	5,272	5,463	0.85 (0.60–1.21)
Additional analyses					
Censoring if dosage change	75	88	7,034	6,966	0.85 (0.62–1.15)
Broader outcome definition^†^	99	145	7,577	7,852	0.70 (0.54–0.91)
Moderate- to high-intensity statin therapy					
Rosuvastatin vs atorvastatin (ref)					
Subgroup analyses					
Male	42	36	2,744	2,773	1.18 (0.76–1.84)
Female	57	43	2,862	2,905	1.34 (0.90–1.99)
40–64 years	59	44	3,448	3,478	1.35 (0.92–2.00)
≥65 years	42	23	2,122	2,141	1.84 (1.11–3.06)
5–10 vs 10–20 mg	95	70	5,426	5,490	1.37 (1.01–1.87)
20–40 vs 40–80 mg	X	5	188	184	0.59 (0.14–2.47)
Sensitivity analyses					
No muscle complaints before CED	88	74	5,179	5,238	1.20 (0.88–1.64)
No use of CYP3A4 inhibiting drugs*	79	63	4,460	4,475	1.26 (0.90–1.75)
Additional analyses					
Censoring if dosage change	96	84	5,395	5,196	1.11 (0.83–1.48)
Broader outcome definition^†^	120	108	5,628	5,658	1.12 (0.86–1.45)
Simvastatin vs atorvastatin (ref)					
Subgroup analyses					
Male	215	152	14,952	15,144	1.43 (1.16–1.76)
Female	279	204	14,204	14,494	1.39 (1.16–1.67)
40–64 years	251	198	16,280	16,638	1.29 (1.07–1.56)
≥65 years	238	159	12,753	12,910	1.52 (1.24–1.85)
40 vs 10 mg	502	352	28,972	29,517	1.45 (1.27–1.66)
80 vs 20 mg	X	X	191	197	1.01 (0.20–5.01)
Sensitivity analyses					
No muscle complaints before CED	413	287	26,347	26,782	1.46 (1.26–1.70)
No use of CYP3A4 inhibiting drugs*	349	290	21,661	21,932	1.22 (1.04–1.42)
Additional analyses					
Censoring if dosage change	468	335	27,920	27,451	1.38 (1.20–1.59)
Broader outcome definition^†^	697	534	29,222	29,708	1.33 (1.18–1.48)

*HR* hazard ratio, *CI* confidence interval, *Ref* reference, *CED* cohort entry date, *CYP3A4* Cytochrome P450 3A4; *X* cell contains <5 patients (not shown owing to ethics regulations to preserve confidentiality)

*The analysis was restricted to patients with no prescription for azole antifungals, macrolide antibiotics, cimetidine, cyclosporine, nefazodone, amiodarone, amlodipine, diltiazem, and verapamil within 180 days before the cohort entry date. We censored patients on the day of a first prescription for one of the drugs during follow-up

†Any recorded Read code for “statin intolerance” qualified as an outcome of interest

#### Moderate- to High-Intensity Statin Therapy

##### Rosuvastatin (hydrophilic) and simvastatin (lipophilic) vs atorvastatin (lipophilic)

In the PS-matched cohort of rosuvastatin vs atorvastatin, IRs of muscular events were 17.8 and 15.2 per 1000 PYs, and in the PS-matched cohort of simvastatin vs atorvastatin, they were 16.5 and 12.4 per 1000 PYs, respectively (Table 3). The overall HR was 1.17 (95% CI 0.88–1.56) for rosuvastatin compared with atorvastatin and 1.33 (95% CI 1.16–1.53) for simvastatin compared with atorvastatin. In both cohorts, the HR was highest during the first 90 days of follow-up, and attenuated towards the null thereafter. The highest HRs, with atorvastatin as the referent, were 1.43 (95% CI 0.82–2.50) for rosuvastatin between days 31–90 of follow-up, and 1.91 (95% CI 1.29–2.81) for simvastatin between days 1–30 of follow-up (Table 4 and Appendix Figure 3). Results of subgroup, sensitivity, and additional analyses were not meaningfully different from the overall result in both cohorts (Table 5). Results from the multivariable regression analyses were consistent with the findings obtained in the PS-matched cohorts (Appendix Table 11).

### Secondary Prevention Group

After PS matching, we observed an overall HR of 1.04 (95% CI 0.68–1.59) for pravastatin when compared with simvastatin, which decreased to 0.89 (95% CI 0.61–1.30) after applying the broader outcome definition (Appendix Table 12). In the PS-matched cohorts of rosuvastatin and simvastatin vs atorvastatin, we found an overall HR of 0.93 (95% CI 0.44–1.99) and 1.43 (95% CI 1.04–1.95), respectively. Broadening the outcome definition resulted in a HR of 1.13 (95% CI 0.58–2.22) for rosuvastatin and a HR of 1.29 (95% CI 1.01–1.66) for simvastatin. The number of events was low in cohorts (iv) and (v) (Appendix Table 12).

#### Discussion

Findings of this large primary care database cohort study do not suggest a systematically reduced risk of muscular events for hydrophilic statins when compared to lipophilic statins at comparable lipid-lowering doses. In the primary prevention study population, results pointed towards a lower muscular risk for pravastatin (hydrophilic) than simvastatin (lipophilic) at doses used for the low-intensity statin therapy, and towards a lower risk of muscular events for atorvastatin (lipophilic) than rosuvastatin (hydrophilic) and simvastatin (lipophilic), when compared at doses used for the moderate- to high-intensity statin therapy. Our results did not reach statistical significance for all comparisons. However, point estimates were furthest from the null within the first 90 days after statin initiation, which provides confidence in the validity of our findings, as statin-associated muscular adverse events predominately occur within the first 6 months after treatment start.^31^

Findings from RCTs comparing statins head-to-head have suggested a comparable tolerability for hydrophilic and lipophilic statins at comparable lipid-lowering doses.^11, 12, 32^ However, the limited sample size of the trials resulted in a low absolute number of muscular events. More importantly, head-to-head RCTs were designed to evaluate statins’ efficacy in the reduction of low-density lipoprotein cholesterol and were not restricted to new statin users. Given that muscular adverse events typically manifest shortly after statin initiation,^31^ inclusion of tolerant prevalent statin users may have resulted in depletion of susceptibles and may have biased safety results towards the null. For instance, the POLARIS trial, a double-blind RCT with a 26-week follow-up, included 871 patients with hypercholesterolemia with or without cardiovascular disease and randomized them to rosuvastatin 40 mg or atorvastatin 80 mg after a dietary run-in period. The study reported 3.0% (*n*=13/432) of drug-related myalgia for rosuvastatin and 3.6% (*n*=16/439) for atorvastatin. However, data on the patient characteristics at study entry are limited and do not provide details on prior statin use.^11^ The same is true for the open-label randomized Dutch DISCOVERY trial, which included hypercholesterolemic patients with or without atherosclerotic disease from 152 primary care physician practices. The authors found that pravastatin 40 mg and simvastatin 20 mg were similarly well tolerated, with 2.4% (*n*= 5/211) of pravastatin users and 1.5% (*n*= 3/194) of simvastatin users having adverse events of myalgia over 12 weeks of follow-up. Although the study reported that around 20% of patients in either treatment group had taken statins in the 4 weeks before enrolment, data on the ever statin use of patients before this date are not available.^12^

The former hypothesis of a systematically reduced muscular risk in association with hydrophilic statins was based on in vitro data demonstrating a lower cytotoxicity on C2C12 myotubes for hydrophilic statins than lipophilic statins,^10^ and in particular on the consideration that hydrophilic statins penetrate skeletal muscles less easily due to lower passive diffusion.^8^ However, the risk of muscular adverse events of statins may depend on different pharmacokinetic processes. For instance, statins are substrates of the organic anion transporting polypeptide (OATP) transport proteins, which are involved in the hepatic and muscular uptake of statins.^33^ It has been discussed that different statins may bind to these transporters with different affinities,^33, 34^ which could eventually lead to different intramuscular drug concentrations and thus to different risk profiles for muscular adverse events. In addition, genetic polymorphism in the *SLCO1B1* gene, which encodes the OATP1B1 hepatic uptake transporter, has been linked to reduced transport activity and increased plasma statin levels.^34, 35^ Levels of simvastatin were found to be particularly affected by the genetic polymorphism,^34^ which may explain the elevated muscular risk for simvastatin observed in the current study. Differences in the metabolism of statins, i.e., statins’ potential for CYP3A4-mediated interactions, probably do not explain current findings given that the results from the sensitivity analysis restricted to patients without use of CYP3A4 inhibiting drugs were comparable to those from the primary analysis.

In this study, we observed an absolute risk of muscular events of 1.3% in all new statin users (1.9% when we included all recordings of statin intolerance). Other observational studies reported higher risks for patients in the routine care setting; the PRIMO study, a countrywide survey including 7,924 patients with hyperlipidemia and high-dosage statin therapy in France, reported an absolute risk of muscular events of 10.5%.^36^ A cohort study performed in 120 Swedish statin initiators and with a follow-up of 1 year reported an absolute risk of 14%.^37^ Differences in findings may be explained by the varying data sources and outcome definitions. We defined muscular events based on Read codes recorded in electronic primary care records, whereas the PRIMO study conducted standardized interviews, and the Swedish cohort study used patient questionnaires to specifically enquire about muscular symptoms.^36, 37^ While the latter approaches may have overestimated the absolute risk of muscular adverse events, we likely underestimated the absolute risk of muscular events, as general practitioners may have modified statin treatment without specifically recording the presumptive adverse event. However, any such outcome misclassification was most likely non-differential and thus, if at all, biased HRs towards unity.^38^

In the comparison of low-intensity statin therapy with pravastatin vs simvastatin, more pravastatin users (13.3%) than simvastatin users (7.4%) were censored due to treatment switch. The imbalance of censoring reasons may be related to the intensified target levels for low-density lipoprotein cholesterol published in the European guidelines in 2003^39^ or the British guidelines in 2005.^40^ Lower therapeutic targets have probably caused general practitioners to intensify statin treatment in patients taking low-intensity statin therapy by either treatment switch or dosage increase, if higher doses were available (8.2% of pravastatin vs 14.9% of simvastatin users were censored due to dosage increase in the additional censoring analysis). When we restricted the comparison of pravastatin vs simvastatin to statin users with a CED between 2000 and 2001, i.e., with end of follow-up before 2003, the comparative muscular risk remained unchanged, but censoring reasons including treatment switch were almost balanced (Δ<2%) between treatment groups.

Some additional limitations need to be considered. First, small sample size in the secondary prevention cohorts prevented calculation of reliable risk estimates. We therefore focused on the study results from the primary prevention cohorts, though we did present all findings for completeness. Second, the dose ratios at which statin doses are comparable in efficacy may vary depending on the literature source. Third, rosuvastatin was the only study drug that was newly licensed in the UK after 1997, i.e., in March 2003.^16^ If bias due to new drug recording in the rosuvastatin group had been present, we would have overestimated the HR of rosuvastatin vs atorvastatin. However, when we restricted the analysis of rosuvastatin vs atorvastatin to users with a CED 2 years after licensing of the former, the HR of muscular events did not change. Fourth, because 98% of our observed events were related to myalgia and not to myositis or rhabdomyolysis, reported HRs primarily refer to the risk of mild statin-associated muscle symptoms. Finally, after PS matching, baseline characteristics were balanced between treatment groups compared, but not necessarily between cohorts. Thus, indirect comparison of the muscular risk of rosuvastatin vs simvastatin, both used for the moderate- to high-intensity statin therapy, was not possible.

In conclusion, this study of UK-based primary health care data does not suggest a systematically reduced risk of muscular events for hydrophilic statins when compared with lipophilic statins at comparable lipid-lowering doses. Low-intensity statin therapy with hydrophilic pravastatin (vs lipophilic simvastatin) and moderate- to high-intensity statin therapy with lipophilic atorvastatin (vs hydrophilic rosuvastatin and lipophilic simvastatin) may be associated with a decreased muscular risk. However, further studies that will replicate our findings are warranted before implications for clinical practice are discussed.

## Supplementary Information

ESM 1 (DOCX 882 kb)

## Declarations

### Additional Statement

This study is based on data from the Clinical Practice Research Datalink obtained under license from the UK Medicines and Healthcare products Regulatory Agency. The data is provided by patients and collected by the NHS as part of their care and support. The interpretation and conclusions contained in this study are those of the authors alone.

## References

[1] National Institute for Health and Care Excellence. Clinical guideline CG181 - Cardiovascular disease: risk assessment and reduction, including lipid modification. https://www.nice.org.uk/guidance/cg181. Published 2014. Accessed November 25, 2019.

[2] Mach F, Baigent C, Catapano AL (2020). 2019 ESC/EAS Guidelines for the management of dyslipidaemias: lipid modification to reduce cardiovascular risk. Eur Heart J..

[3] Stroes ES, Thompson PD, Corsini A (2015). Statin-associated muscle symptoms: impact on statin therapy - European Atherosclerosis Society Consensus Panel Statement on Assessment, Aetiology and Management. Eur Heart J..

[4] Joy TR, Hegele RA (2009). Narrative review: statin-related myopathy. Ann Intern Med..

[5] Mammen AL, Amato AA (2010). Statin myopathy: A review of recent progress. Curr Opin Rheumatol..

[6] Auer J, Sinzinger H, Franklin B, Berent R (2016). Muscle- and skeletal-related side-effects of statins: tip of the iceberg?. Eur J Prev Cardiol..

[7] Chatzizisis YS, Koskinas KC, Misirli G, Vaklavas C, Hatzitolios A, Giannoglou GD (2010). Risk factors and drug interactions predisposing to statin-induced myopathy: implications for risk assessment, prevention and treatment. Drug Saf..

[8] Bitzur R, Cohen H, Kamari Y, Harats D (2013). Intolerance to statins: Mechanisms and management. Diabetes Care..

[9] Turner RM, Pirmohamed M (2019). Statin-Related Myotoxicity: A Comprehensive Review of Pharmacokinetic, Pharmacogenomic and Muscle Components. J Clin Med..

[10] Bonifacio A, Sanvee GM, Bouitbir J, Krähenbühl S (1853). The AKT/mTOR signaling pathway plays a key role in statin-induced myotoxicity. Biochim Biophys Acta..

[11] Leiter LA, Rosenson RS, Stein E (2007). Efficacy and safety of rosuvastatin 40 mg versus atorvastatin 80 mg in high-risk patients with hypercholesterolemia: Results of the POLARIS study. Atherosclerosis..

[12] Bots AF, Kastelein JJ (2005). Discovery Netherlands Investigators. Achieving lipid goals in real life: The Dutch DISCOVERY study. Int J Clin Pract..

[13] Ghosh RE, Crellin E, Beatty S, Donegan K, Myles P, Williams R (2019). How Clinical Practice Research Datalink data are used to support pharmacovigilance. Ther Adv Drug Saf..

[14] Strom BL, Kimmel SE, Hennessy S (2020). Electronic Health Record Databases. Pharmacoepidemiology.

[15] Herrett E, Gallagher AM, Bhaskaran K (2015). Data Resource Profile: Clinical Practice Research Datalink (CPRD). Int J Epidemiol..

[16] Electronic medicines compendium. Crestor 10 mg film-coated tablets - Summary of Product Characteristics. https://www.medicines.org.uk/emc/product/7559/smpc. Accessed February 11, 2020.

[17] Vinogradova Y, Coupland C, Brindle P, Hippisley-Cox J (2016). Discontinuation and restarting in patients on statin treatment: Prospective open cohort study using a primary care database. BMJ..

[18] Mancini GB, Baker S, Bergeron J (2016). Diagnosis, Prevention, and Management of Statin Adverse Effects and Intolerance: Canadian Consensus Working Group Update (2016). Can J Cardiol..

[19] Dalakas MC (2009). Toxic and drug-induced myopathies. J Neurol Neurosurg Psychiatry..

[20] Laufs U, Scharnagl H, Halle M, Windler E, Endres M, März W (2015). Treatment Options for Statin-Associated Muscle Symptoms. Dtsch Arztebl Int..

[21] Clegg A, Bates C, Young J (2016). Development and validation of an electronic frailty index using routine primary care electronic health record data. Age Ageing..

[22] Patorno E, Grotta A, Bellocco R, Schneeweiss S (2013). Propensity score methodology for confounding control in health care utilization databases. Epidemiol Biostat Public Health..

[23] Austin PC (2011). An introduction to propensity score methods for reducing the effects of confounding in observational studies. Multivariate Behav Res..

[24] Department of Health and Social Care. National Service Framework for Coronary Heart Disease. Modern Standards and Service Models. London: Department of Health and Social Care; 2000. https://www.gov.uk/government/publications/quality-standards-for-coronary-heart-disease-care.

[25] Cooper A, O’Flynn N (2008). Guideline Development Group. Risk assessment and lipid modification for primary and secondary prevention of cardiovascular disease: Summary of NICE guidance. BMJ..

[26] Helin-Salmivaara A, Lavikainen P, Aarnio E, Huupponen R, Korhonen MJ (2014). Sequential cohort design applying propensity score matching to analyze the comparative effectiveness of atorvastatin and simvastatin in preventing cardiovascular events. PLoS One..

[27] Rubin DB (2001). Using propensity scores to help design observational studies: Application to the tobacco litigation. Health Services & Outcomes Research Methodology..

[28] Austin PC (2009). Using the standardized difference to compare the prevalence of a binary variable between two groups in observational research. Commun Stat Simul Comput..

[29] Sathasivam S (2012). Statin induced myotoxicity. Eur J Intern Med..

[30] Rowan CG, Brunelli SM, Munson J (2012). Clinical importance of the drug interaction between statins and CYP3A4 inhibitors: a retrospective cohort study in The Health Improvement Network. Pharmacoepidemiol Drug Saf..

[31] Banach M, Rizzo M, Toth PP (2015). Statin intolerance - An attempt at a unified definition. Position paper from an International Lipid Expert Panel. Arch Med Sci..

[32] Wlodarczyk J, Sullivan D, Smith M (2008). Comparison of benefits and risks of rosuvastatin versus atorvastatin from a meta-analysis of head-to-head randomized controlled trials. Am J Cardiol..

[33] Bouitbir J, Sanvee GM, Panajatovic MV, Singh F, Krähenbühl S (2020). Mechanisms of statin-associated skeletal muscle-associated symptoms. Pharmacol Res..

[34] Niemi M (2010). Transporter pharmacogenetics and statin toxicity. Clin Pharmacol Ther..

[35] Moßhammer D, Schaeffeler E, Schwab M, Mörike K (2014). Mechanisms and assessment of statin-related muscular adverse effects. Br J Clin Pharmacol..

[36] Bruckert E, Hayem G, Dejager S, Yau C, Bégaud B (2005). Mild to moderate muscular symptoms with high-dosage statin therapy in hyperlipidemic patients - The PRIMO study. Cardiovasc Drugs Ther..

[37] Skilving I, Eriksson M, Rane A, Ovesjö ML (2016). Statin-induced myopathy in a usual care setting—a prospective observational study of gender differences. Eur J Clin Pharmacol..

[38] Funk MJ, Landi SN (2014). Misclassification in administrative claims data: quantifying the impact on treatment effect estimates. Curr Epidemiol Rep..

[39] De Backer G, Ambrosioni E, Borch-Johnsen K (2003). European guidelines on cardiovascular disease prevention in clinical practice. Third Joint Task Force of European and Other Societies on Cardiovascular Disease Prevention in Clinical Practice. Eur Heart J..

[40] British Cardiac Society, British Hypertension Society, Diabetes UK, HEART UK, Primary Care Cardiovascular Society, Stroke Association (2005). JBS 2: Joint British Societies’ guidelines on prevention of cardiovascular disease in clinical practice. Heart.

