# Supplementary material for: The Risk of Muscular Events Among New Users of Hydrophilic and Lipophilic Statins: an Observational Cohort Study
Source: J Gen Intern Med. 2021 Mar 9;36(9):2639–47. doi: 10.1007/s11606-021-06651-6 (PMC8390626; doi:10.1007/s11606-021-06651-6)

**APPENDIX MATERIAL**

| **Appendix Table 1. List of Read codes for the outcome of a muscular event** | | |
| --- | --- | --- |
| I. |  |  |
| 1DCC.00 | Aching muscles |  |
| N241012 | Muscle pain |  |
| N241000 | Myalgia unspecified |  |
| R01z200 | Musculoskeletal pain |  |
| N241z00 | Myalgia or myositis NOS |  |
| N241.00 | Myalgia and myositis unspecified |  |
| N241100 | Myositis unspecified |  |
| Nyu8000 | Other myositis |  |
| F39W.00 | Inflammatory myopathy; not elsewhere classified |  |
| N233300 | Rhabdomyolysis |  |
| K045.00 | Acute renal failure due to non-traumatic rhabdomyolysis |  |
| SK08.00 | Acute renal failure due to rhabdomyolysis |  |
| R113.00 | Myoglobinuria |  |
| F394000 | Drug-induced myopathy |  |
| F394.00 | Toxic myopathy |  |
| F397.00 | Proximal myopathy |  |
| Fyu8200 | Other specified myopathies |  |
| Nyu8.00 | Disorders of muscles^*^ |  |
| Nyu8500 | Other specified disorders of muscle^*^ |  |
| Nyu8B00 | Disorder of muscle; unspecified^*^ |  |
| N233.00 | Other specific muscle disorder^*^ |  |
| N233z00 | Other specific muscle disorder^*^ |  |
| II.^†^ |  |  |
| 8I76.00 | Statin not tolerated |  |
| U60CA00 | Statin causing adverse effect in therapeutic use |  |
| TJC2400 | Adverse reaction to simvastatin |  |
| U60C615 | Adverse reaction to simvastatin |  |
| TJC2500 | Adverse reaction to pravastatin |  |
| U60C616 | Adverse reaction to pravastatin |  |
| ^*^ These 5 Read codes resulted in one outcome event in total.  ^†^ If followed by a Read code listed under I. within 90 days. | | |

| **Appendix Table 2. Set of baseline covariates^*^** | |  |
| --- | --- | --- |
| **Demographics**  Age  Sex  **Lifestyle factors**  *last record entry before the cohort entry date*  Smoking status  current vs former or never  Alcohol consumption  ≤ vs >14 units of alcohol per week  **Comorbidities**  *recorded at any time before the cohort entry date*  Diabetes mellitus^†^  Chronic kidney disease^‡^  Severe liver impairment  Hypo-/ Hyperthyroidism  Pre-existing muscle complaints  Musculoskeletal injuries  Indicators for health status / frailty  Myocardial infarction^§^  Ischemic stroke^§^  Hemorrhagic stroke  Hypertension  Heart failure  Atrial fibrillation  Ischemic heart disease  Peripheral arterial disease  Hyperlipidemia^‖^  Rheumatoid Arthritis  Osteoarthritis  Chronic obstructive pulmonary disease  Macular degeneration  *recorded in the 3 years before the cohort entry date*  Falls  Pressure ulcer  Incontinence  Peripheral venous thrombosis  Pneumonia  Dysphagia  Anemia | **Comedication**  *recorded in the 180 days before the cohort entry date* Fibrates  Drugs causing myopathy  Amiodarone  Systemic corticosteroids  Antipsychotics (i.e. clozapine, risperidone,  olanzapine, loxapine, haloperidol)  Indicators for health status / frailty  H_2_-receptor antagonists  Benzodiazepine  Number of cardiovascular drug classes  0, 1-3, or 4-10 drug classes, including  ACE inhibitors  Angiotensin II receptor blockers  Beta blockers  Calcium channel blockers  Coronary vasodilators  Diuretics  Antiarrhythmics  Antiplatelet agents  Vitamin K antagonists  Other lipid-lowering agents (ezetimibe,  fibrates, nicotinic acid and derivatives, bile  acid sequestrants, omega-3 fatty acid  compounds)  **Health care utilization**  *in the year before the cohort entry date*  Number of general practitioner visits  *recorded in the 3 years before the cohort entry date*  Hospitalization  **Other variables**  *last record entry before the cohort entry date*  Obesity  body mass index < 30 vs ≥ 30 kg/m^2^  *at the date of cohort entry*  Initially prescribed daily statin dose | |
| *ACE* angiotensin-converting enzyme | |  |
| ^*^ The following covariates were not included in the statistical analyses due to a prevalence of <0.5% among eligible statin initiators, irrespective of statin type: comorbidities: hypo-/hyperparathyroidism, vitamin D deficiency, Cushing’s syndrome, adrenal insufficiency, lupus erythematosus, scleroderma, Sjoegren’s syndrome, mixed connective disease, sarcoidosis, polyarteritis nodosa, and Down syndrome; comedication: niacin, D-penicillamine, colchicine, hydroxy-/chloroquine, interferons, and fusidic acid.  ^†^ Defined as either a Read code for diabetes mellitus or a recorded prescription for an antidiabetic drug ≤180 days before the cohort entry date.  ^‡^ Defined as either a Read code for chronic kidney disease or two successive glomerular filtration rate measurements <60 ml/min, separated by ≥90 days, with the first measurement being the closest before the cohort entry date.  ^§^ Only assessed in the secondary prevention cohorts.  ^‖^ Defined as either a Read code for hyperlipidemia or a last recorded low-density lipoprotein level of ≥3 mmol/L before the cohort entry date. | |  |

| **Appendix Table 3. Detailed information on the subgroup analyses, sensitivity analyses, and additional analyses** | |
| --- | --- |
| **Subgroup analyses** | We performed subgroup analyses by   - sex, - age, i.e. </≥ 65 years, - initial daily statin dose, i.e. ≤/> 20 mg of pravastatin; ≤/> 10 mg of rosuvastatin; ≤/> 40 mg of simvastatin; equivalent doses of the comparator statins: ≤/> 10 mg of simvastatin; ≤/> 20 mg of atorvastatin; ≤/> 10 mg of atorvastatin.   (primary prevention cohorts) |
| **Sensitivity analysis 1:**  **No muscle complaints before the cohort entry date** | We performed a sensitivity analysis restricted to patients with no recording of muscle complaints (including myalgia, muscle pain, muscle ache, spasm, or cramps) at any time before the cohort entry date.  (primary prevention cohorts) |
| **Sensitivity analysis 2:**  **No use of CYP3A4 inhibiting drugs** | We performed a sensitivity analysis restricted to patients with no prescription for a drug that inhibits the enzyme CYP3A4 (i.e. azole antifungals, macrolide antibiotics, cimetidine, cyclosporine, nefazodone, amiodarone, amlodipine, diltiazem, and verapamil), within 6 months before the cohort entry date (censoring on the date of a first prescription during follow-up). Simvastatin and atorvastatin are metabolized by CYP3A4, and their serum concentrations may be increased if co-prescribed with CYP3A4 inhibiting drugs.  (primary prevention cohorts) |
| **Additional analysis 1:**  **Censoring if dosage change** | We additionally censored patients on the date of dosage change (prescription for a dose of the study drug other than the initially prescribed one). If a Read code for a muscular event was recorded within 90 days after dosage reduction, we considered the date of dosage reduction as the date when an event occurred.  (primary prevention cohorts) |
| **Additional analysis 2:**  **Broader outcome definition** | We performed an analysis applying a broader outcome definition, in which any recorded Read code for ‘statin intolerance’ qualified as an outcome of interest.  (primary and secondary prevention cohorts) |
| **Additional analysis 3:**  **Multivariable logistic regression analyses** | We repeated our analyses in the cohorts before propensity score matching, using multivariable logistic regression and adjusting for all baseline covariates as well as for calendar year of cohort entry.  (primary and secondary prevention cohorts) |
| *CYP3A4* Cytochrome P450 3A4 |  |

| **Appendix Table 4. Baseline covariates of users of simvastatin 40-80 mg and atorvastatin 10-20 mg (moderate- to high-intensity statin therapy) in the primary prevention cohort before and after propensity score matching** | | | | | | | | |
| --- | --- | --- | --- | --- | --- | --- | --- | --- |
|  |  | **Cohort before**  **propensity score matching** | |  |  | **Cohort after**  **propensity score matching** | |  |
| **Covariate** |  | **Simvastatin**  **N = 161,572** | **Atorvastatin**  **N = 101,359** | **Absolute standardized difference (%)** |  | **Simvastatin N = 37,743** | **Atorvastatin**  **N = 37,743** | **Absolute standardized difference (%)** |
| Age [years], mean (SD) |  | 61.0 (9.7) | 61.8 (9.8) | -8.4 |  | 62.0 (9.8) | 62.0 (9.9) | -0.4 |
| Male, n (%) |  | 88,473 (54.8) | 49,358 (48.7) | 12.2 |  | 19,176 (50.8) | 19,152 (50.7) | 0.1 |
| Current smoker^*^, n (%) |  | 36,067 (22.3) | 18,994 (18.7) | 8.9 |  | 7,314 (19.4) | 7,222 (19.1) | 0.6 |
| >14 units of alcohol per week^*^, n (%) |  | 19,569 (12.1) | 9,995 (9.9) | 7.2 |  | 3,719 (9.9) | 3,784 (10.0) | -0.6 |
| Obesity^*^, n (%) |  | 55,138 (34.1) | 32,248 (31.8) | 4.9 |  | 12,349 (32.7) | 12,341 (32.7) | 0.0 |
| Comorbidities, n (%) - at any time before the cohort entry date, if not specified otherwise | | | | |  |  |  |  |
| Hyperlipidemia |  | 113,122 (70.0) | 68,675 (67.8) | 4.9 |  | 24,565 (65.1) | 24,664 (65.3) | -0.6 |
| Diabetes mellitus |  | 32,691 (20.2) | 25,456 (25.1) | -11.7 |  | 9,892 (26.2) | 9,896 (26.2) | -0.0 |
| Hypertension |  | 74,186 (45.9) | 50,638 (50.0) | -8.1 |  | 19,159 (50.8) | 19,168 (50.8) | -0.0 |
| Heart failure |  | 2,087 (1.3) | 1,807 (1.8) | -4.0 |  | 734 (1.9) | 768 (2.0) | -0.6 |
| Atrial fibrillation |  | 5,302 (3.3) | 3,945 (3.9) | -3.3 |  | 1,539 (4.1) | 1,553 (4.1) | -0.2 |
| Ischemic heart disease |  | 13,720 (8.5) | 12,222 (12.1) | -11.8 |  | 4,734 (12.5) | 4,777 (12.7) | -0.3 |
| Peripheral arterial disease |  | 3,628 (2.2) | 2,779 (2.7) | -3.2 |  | 1,016 (2.7) | 1,019 (2.7) | -0.0 |
| Hemorrhagic stroke |  | 1,098 (0.7) | 562 (0.6) | 1.6 |  | 283 (0.7) | 273 (0.7) | 0.3 |
| Chronic kidney disease |  | 12,667 (7.8) | 7,202 (7.1) | 2.8 |  | 2,941 (7.8) | 3,035 (8.0) | -0.9 |
| Severe liver impairment |  | 196 (0.1) | 135 (0.1) | -0.3 |  | 59 (0.2) | 65 (0.2) | -0.4 |
| Hypothyroidism |  | 11,460 (7.1) | 7,550 (7.4) | -1.4 |  | 2,948 (7.8) | 2,886 (7.6) | 0.6 |
| Hyperthyroidism |  | 2,620 (1.6) | 1,721 (1.7) | -0.6 |  | 695 (1.8) | 682 (1.8) | 0.3 |
| Rheumatoid Arthritis |  | 2,622 (1.6) | 1,658 (1.6) | -0.1 |  | 742 (2.0) | 754 (2.0) | -0.2 |
| Osteoarthritis |  | 31,489 (19.5) | 19,344 (19.1) | 1.0 |  | 7,490 (19.8) | 7,455 (19.8) | 0.2 |
| Pre-existing muscle complaints |  | 16,071 (9.9) | 9,620 (9.5) | 1.5 |  | 3,772 (10.0) | 3,716 (9.8) | 0.5 |
| Musculoskeletal injuries |  | 57,913 (35.8) | 33,078 (32.6) | 6.8 |  | 12,734 (33.7) | 12,651 (33.5) | 0.5 |
| Chronic obstructive pulmonary disease |  | 7,205 (4.5) | 3,926 (3.9) | 2.9 |  | 1,585 (4.2) | 1,571 (4.2) | 0.2 |
| Macular degeneration |  | 1076 (0.7) | 686 (0.7) | -0.1 |  | 278 (0.7) | 293 (0.8) | -0.5 |
| Falls^†^ |  | 4,673 (2.9) | 2,780 (2.7) | 0.9 |  | 1,201 (3.2) | 1,149 (3.0) | 0.8 |
| Pressure ulcer^†^ |  | 552 (0.3) | 387 (0.4) | -0.7 |  | 154 (0.4) | 160 (0.4) | -0.2 |
| Incontinence^†^ |  | 1,541 (1.0) | 959 (0.9) | 0.1 |  | 373 (1.0) | 396 (1.0) | -0.6 |
| Peripheral venous thrombosis^†^ |  | 1,905 (1.2) | 1,299 (1.3) | -0.9 |  | 517 (1.4) | 497 (1.3) | 0.5 |
| Pneumonia^†^ |  | 938 (0.6) | 542 (0.5) | 0.6 |  | 213 (0.6) | 233 (0.6) | -0.7 |
| Dysphagia^†^ |  | 1,026 (0.6) | 544 (0.5) | 1.3 |  | 233 (0.6) | 230 (0.6) | 0.1 |
| Anemia^†^ |  | 2,976 (1.8) | 1,840 (1.8) | 0.2 |  | 773 (2.0) | 763 (2.0) | 0.2 |
| Comedication, n (%) - in the 180 days before the cohort entry date | | | |  |  |  |  |  |
| Fibrates |  | 697 (0.4) | 1,433 (1.4) | -10.3 |  | 347 (0.9) | 357 (0.9) | -0.3 |
| Amiodarone |  | 513 (0.3) | 559 (0.6) | -3.6 |  | 231 (0.6) | 221 (0.6) | 0.3 |
| Systemic corticosteroids |  | 5,773 (3.6) | 3,774 (3.7) | -0.8 |  | 1,441 (3.8) | 1,438 (3.8) | 0.0 |
| Antipsychotics |  | 1,219 (0.8) | 555 (0.5) | 2.6 |  | 207 (0.5) | 197 (0.5) | 0.4 |
| H_2_-receptor antagonists |  | 3,870 (2.4) | 3,605 (3.6) | -6.8 |  | 1,288 (3.4) | 1,258 (3.3) | 0.4 |
| Benzodiazepines |  | 9,529 (5.9) | 7,019 (6.9) | -4.2 |  | 2,658 (7.0) | 2,596 (6.9) | 0.6 |
| Number of cardiovascular drug classes |  |  |  |  |  |  |  |  |
| 0 |  | 52,285 (32.4) | 30,680 (30.3) | 4.5 |  | 10,108 (26.8) | 10,112 (26.8) | -0.0 |
| 1 to 3 |  | 93,774 (58.0) | 59,730 (58.9) | -1.8 |  | 22,878 (60.6) | 22,836 (60.5) | 0.2 |
| 4 to 10 |  | 15,513 (9.6) | 10,949 (10.8) | -4.0 |  | 4,757 (12.6) | 4,795 (12.7) | -0.3 |
| Number of general practitioner visits^‡^, mean (SD) |  | 19.8 (13.0) | 20.2 (12.9) | -3.7 |  | 21.0 (13.2) | 20.9 (13.2) | 0.4 |
| Hospitalization^†^, n (%) |  | 54,000 (33.4) | 30,092 (29.7) | 8.0 |  | 11,719 (31.1) | 11,623 (30.8) | 0.6 |
| Daily statin dose [mg], n (%) |  |  |  |  |  |  |  |  |
| 40 (simvastatin) vs 10 (atorvastatin) |  | 161,318 (99.8) | 65,325 (64.4) | 104.2 |  | 37,490 (99.3) | 37,490 (99.3) | 0.0 |
| 80 (simvastatin) vs 20 (atorvastatin) |  | 254 (0.2) | 36,034 (35.6) | -104.2 |  | 253 (0.7) | 253 (0.7) | 0.0 |
| Cohort entry date, n (%) |  |  |  |  |  |  |  |  |
| 2000-2001 |  | 417 (0.3) | 13,531 (13.3) | -53.8 |  | 416 (1.1) | 416 (1.1) | 0.0 |
| 2002-2003 |  | 7,045 (4.4) | 23,137 (22.8) | -56.0 |  | 7,045 (18.7) | 7,045 (18.7) | 0.0 |
| 2004-2005 |  | 16,211 (10.0) | 24,286 (24.0) | -37.7 |  | 14,903 (39.5) | 14,903 (39.5) | 0.0 |
| 2006-2007 |  | 31,460 (19.5) | 5,770 (5.7) | 42.5 |  | 3,744 (9.9) | 3,744 (9.9) | 0.0 |
| 2008-2009 |  | 40,161 (24.9) | 1,414 (1.4) | 74.1 |  | 845 (2.2) | 845 (2.2) | 0.0 |
| 2010-2011 |  | 32,818 (20.3) | 842 (0.8) | 66.8 |  | 479 (1.3) | 479 (1.3) | 0.0 |
| 2012-2013 |  | 23,541 (14.6) | 6,381 (6.3) | 27.3 |  | 3,793 (10.0) | 3,793 (10.0) | 0.0 |
| 2014-2015 |  | 7,901 (4.9) | 13,652 (13.5) | -30.0 |  | 4,755 (12.6) | 4,755 (12.6) | 0.0 |
| 2016-2017 |  | 2,018 (1.2) | 12,346 (12.2) | -44.8 |  | 1,763 (4.7) | 1,763 (4.7) | 0.0 |
| *SD* standard deviation  ^*^ Last record before the cohort entry date.  ^†^ Assessed in the 3 years before the cohort entry date.  ^‡^ Assessed in the 1 year before the cohort entry date. | | | | | | | | |

| **Appendix Table 5. Baseline covariates of users of pravastatin 20-40 mg and simvastatin 10-20 mg (low-intensity statin therapy) in the secondary prevention cohort before and after propensity score matching** | | | | | | | | |
| --- | --- | --- | --- | --- | --- | --- | --- | --- |
|  |  | **Cohort before**  **propensity score matching** | |  |  | **Cohort after**  **propensity score matching** | |  |
| **Covariate** |  | **Pravastatin**  **N = 4,139** | **Simvastatin**  **N = 24,836** | **Absolute standardized difference (%)** |  | **Pravastatin**  **N = 4,121** | **Simvastatin**  **N = 4,121** | **Absolute standardized difference (%)** |
| Age [years], mean (SD) |  | 65.9 (9.7) | 67.3 (9.2) | -14.7 |  | 65.9 (9.7) | 65.9 (9.7) | 0.3 |
| Male, n (%) |  | 2,582 (62.4) | 14,717 (59.3) | 6.4 |  | 2,574 (62.5) | 2,619 (63.6) | -2.3 |
| Current smoker^*^, n (%) |  | 1,050 (25.4) | 5,478 (22.1) | 7.8 |  | 1,048 (25.4) | 1,017 (24.7) | 1.7 |
| >14 units of alcohol per week^*^, n (%) |  | 335 (8.1) | 2,128 (8.6) | -1.7 |  | 334 (8.1) | 313 (7.6) | 1.9 |
| Obesity^*^, n (%) |  | 835 (20.2) | 5,049 (20.3) | -0.4 |  | 830 (20.1) | 847 (20.6) | -1.0 |
| Comorbidities, n (%) - at any time before the cohort entry date, if not specified otherwise | | | | |  |  |  |  |
| Hyperlipidemia |  | 1,452 (35.1) | 11,147 (44.9) | -20.1 |  | 1,443 (35.0) | 1,427 (34.6) | 0.8 |
| Diabetes mellitus |  | 605 (14.6) | 3,353 (13.5) | 3.2 |  | 603 (14.6) | 607 (14.7) | -0.3 |
| Hypertension |  | 1,946 (47.0) | 12,331 (49.6) | -5.3 |  | 1,939 (47.1) | 1,963 (47.6) | -1.2 |
| Heart failure |  | 353 (8.5) | 1,600 (6.4) | 7.9 |  | 349 (8.5) | 348 (8.4) | 0.1 |
| Atrial fibrillation |  | 449 (10.8) | 2,033 (8.2) | 9.1 |  | 443 (10.7) | 429 (10.4) | 1.1 |
| Ischemic heart disease |  | 1,912 (46.2) | 9,147 (36.8) | 19.1 |  | 1,904 (46.2) | 1877 (45.5) | 1.3 |
| Peripheral arterial disease |  | 258 (6.2) | 1,352 (5.4) | 3.4 |  | 258 (6.3) | 249 (6.0) | 0.9 |
| Hemorrhagic stroke |  | 85 (2.1) | 495 (2.0) | 0.4 |  | 84 (2.0) | 71 (1.7) | 2.3 |
| Ischemic stroke |  | 1,912 (46.2) | 15,252 (61.4) | -30.9 |  | 1,904 (46.2) | 1,865 (45.3) | 1.9 |
| Myocardial infarction |  | 2,438 (58.9) | 10,502 (42.3) | 33.7 |  | 2,427 (58.9) | 2,441 (59.2) | -0.7 |
| Chronic kidney disease |  | 269 (6.5) | 2,486 (10.0) | -12.8 |  | 264 (6.4) | 246 (6.0) | 1.8 |
| Severe liver impairment |  | 5 (0.1) | 48 (0.2) | -1.8 |  | 5 (0.1) | 5 (0.1) | 0.0 |
| Hypothyroidism |  | 218 (5.3) | 1,614 (6.5) | -5.2 |  | 215 (5.2) | 202 (4.9) | 1.4 |
| Hyperthyroidism |  | 57 (1.4) | 440 (1.8) | -3.2 |  | 56 (1.4) | 58 (1.4) | -0.4 |
| Rheumatoid Arthritis |  | 96 (2.3) | 519 (2.1) | 1.6 |  | 95 (2.3) | 92 (2.2) | 0.5 |
| Osteoarthritis |  | 881 (21.3) | 5,442 (21.9) | -1.5 |  | 877 (21.3) | 844 (20.5) | 2.0 |
| Pre-existing muscle complaints |  | 373 (9.0) | 2,439 (9.8) | -2.8 |  | 371 (9.0) | 371 (9.0) | 0.0 |
| Musculoskeletal injuries |  | 1,169 (28.2) | 7,311 (29.4) | -2.6 |  | 1,164 (28.2) | 1,111 (27.0) | 2.9 |
| Chronic obstructive pulmonary disease |  | 264 (6.4) | 1,644 (6.6) | -1.0 |  | 263 (6.4) | 249 (6.0) | 1.4 |
| Macular degeneration |  | 49 (1.2) | 351 (1.4) | -2.0 |  | 48 (1.2) | 59 (1.4) | -2.4 |
| Falls^†^ |  | 159 (3.8) | 1,220 (4.9) | -5.2 |  | 157 (3.8) | 155 (3.8) | 0.3 |
| Pressure ulcer^†^ |  | 30 (0.7) | 167 (0.7) | 0.6 |  | 30 (0.7) | 34 (0.8) | -1.1 |
| Incontinence^†^ |  | 55 (1.3) | 381 (1.5) | -1.7 |  | 55 (1.3) | 44 (1.1) | 2.5 |
| Peripheral venous thrombosis^†^ |  | 76 (1.8) | 424 (1.7) | 1.0 |  | 76 (1.8) | 79 (1.9) | -0.5 |
| Pneumonia^†^ |  | 39 (0.9) | 247 (1.0) | -0.5 |  | 39 (0.9) | 40 (1.0) | -0.2 |
| Dysphagia^†^ |  | 33 (0.8) | 212 (0.9) | -0.6 |  | 33 (0.8) | 22 (0.5) | 3.3 |
| Anemia^†^ |  | 114 (2.8) | 739 (3.0) | -1.3 |  | 113 (2.7) | 130 (3.2) | -2.4 |
| Comedication, n (%) - in the 180 days before the cohort entry date | | | |  |  |  |  |  |
| Fibrates |  | 63 (1.5) | 268 (1.1) | 3.9 |  | 62 (1.5) | 65 (1.6) | -0.6 |
| Amiodarone |  | 121 (2.9) | 542 (2.2) | 4.7 |  | 120 (2.9) | 102 (2.5) | 2.7 |
| Systemic corticosteroids |  | 192 (4.6) | 960 (3.9) | 3.8 |  | 190 (4.6) | 171 (4.1) | 2.3 |
| Antipsychotics |  | 15 (0.4) | 151 (0.6) | -3.5 |  | 15 (0.4) | 12 (0.3) | 1.3 |
| H_2_-receptor antagonists |  | 352 (8.5) | 1,430 (5.8) | 10.7 |  | 352 (8.5) | 367 (8.9) | -1.3 |
| Benzodiazepines |  | 452 (10.9) | 2,269 (9.1) | 5.9 |  | 449 (10.9) | 434 (10.5) | 1.2 |
| Number of cardiovascular drug classes |  |  |  |  |  |  |  |  |
| 0 |  | 130 (3.1) | 1,388 (5.6) | -12.0 |  | 129 (3.1) | 120 (2.9) | 1.3 |
| 1 to 3 |  | 2,300 (55.6) | 16,207 (65.3) | -19.9 |  | 2,288 (55.5) | 2,301 (55.8) | -0.6 |
| 4 to 10 |  | 1,709 (41.3) | 7,241 (29.2) | 25.6 |  | 1,704 (41.3) | 1,700 (41.3) | 0.2 |
| Number of general practitioner visits^‡^, mean (SD) |  | 20.5 (13.6) | 21.7 (14.0) | -9.0 |  | 20.4 (13.5) | 20.4 (13.7) | -0.2 |
| Hospitalization^†^, n (%) |  | 1,548 (37.4) | 8,440 (34.0) | 7.1 |  | 1,535 (37.2) | 1,548 (37.6) | -0.7 |
| Daily statin dose [mg], n (%) |  |  |  |  |  |  |  |  |
| 20 (pravastatin) vs 10 (simvastatin) |  | 1,348 (32.6) | 8,349 (33.6) | -2.2 |  | 1,338 (32.5) | 1,329 (32.2) | 0.5 |
| 40 (pravastatin) vs 20 (simvastatin) |  | 2,791 (67.4) | 16,487 (66.4) | 2.2 |  | 2,783 (67.5) | 2,792 (67.8) | -0.5 |
| Cohort entry date, n (%) |  |  |  |  |  |  |  |  |
| 2000-2001 |  | 1,617 (39.1) | 6,222 (25.1) | 30.4 |  | 1,617 (39.2) | 1,617 (39.2) | 0.0 |
| 2002-2003 |  | 1,821 (44.0) | 6,909 (27.8) | 34.2 |  | 1,821 (44.2) | 1,821 (44.2) | 0.0 |
| 2004-2005 |  | 500 (12.1) | 5,975 (24.1) | -31.5 |  | 500 (12.1) | 500 (12.1) | 0.0 |
| 2006-2007 |  | 72 (1.7) | 2,983 (12.0) | -41.5 |  | 71 (1.7) | 71 (1.7) | 0.0 |
| 2008-2009 |  | 47 (1.1) | 1,257 (5.1) | -22.8 |  | 44 (1.1) | 44 (1.1) | 0.0 |
| 2010-2011 |  | 49 (1.2) | 602 (2.4) | -9.3 |  | 44 (1.1) | 44 (1.1) | 0.0 |
| 2012-2013 |  | 22 (0.5) | 519 (2.1) | -13.7 |  | 21 (0.5) | 21 (0.5) | 0.0 |
| 2014-2015 |  | 7 (0.2) | 289 (1.2) | -12.2 |  | X | X | X |
| 2016-2017 |  | X | 80 (0.3) | X |  | 0 | 0 | 0.0 |
| *SD* standard deviation; *X* cell contains <5 patients (not shown owing to ethics regulations to preserve confidentiality)  ^*^ Last record before the cohort entry date.  ^†^ Assessed in the 3 years before the cohort entry date.  ^‡^ Assessed in the 1 year before the cohort entry date | | | | | | | | |

| **Appendix Table 6. Baseline covariates of users of rosuvastatin 5-40 mg and atorvastatin 10-80 mg (moderate- to high-intensity statin therapy) in the secondary prevention cohort before and after propensity score matching** | | | | | | | | |
| --- | --- | --- | --- | --- | --- | --- | --- | --- |
|  |  | **Cohort before**  **propensity score matching** | |  |  | **Cohort after**  **propensity score matching** | |  |
| **Covariate** |  | **Rosuvastatin**  **N = 891** | **Atorvastatin**  **N = 18,000** | **Absolute standardized difference (%)** |  | **Rosuvastatin**  **N = 836** | **Atorvastatin**  **N = 836** | **Absolute standardized difference (%)** |
| Age [years], mean (SD) |  | 66.6 (9.6) | 63.4 (10.5) | 31.4 |  | 66.6 (9.5) | 66.7 (9.6) | -1.5 |
| Male, n (%) |  | 516 (57.9) | 11,536 (64.1) | -12.7 |  | 482 (57.7) | 461 (55.1) | 5.1 |
| Current smoker^*^, n (%) |  | 209 (23.5) | 4,791 (26.6) | -7.3 |  | 198 (23.7) | 200 (23.9) | -0.6 |
| >14 units of alcohol per week^*^, n (%) |  | 79 (8.9) | 1,847 (10.3) | -4.7 |  | 72 (8.6) | 67 (8.0) | 2.2 |
| Obesity^*^, n (%) |  | 223 (25.0) | 4,407 (24.5) | 1.3 |  | 206 (24.6) | 191 (22.8) | 4.2 |
| Comorbidities, n (%) - at any time before the cohort entry date, if not specified otherwise | | | | |  |  |  |  |
| Hyperlipidemia |  | 460 (51.6) | 8,312 (46.2) | 10.9 |  | 428 (51.2) | 426 (51.0) | 0.5 |
| Diabetes mellitus |  | 112 (12.6) | 1,807 (10.0) | 8.0 |  | 105 (12.6) | 104 (12.4) | 0.4 |
| Hypertension |  | 447 (50.2) | 6,939 (38.6) | 23.5 |  | 421 (50.4) | 430 (51.4) | -2.2 |
| Heart failure |  | 51 (5.7) | 961 (5.3) | 1.7 |  | 45 (5.4) | 51 (6.1) | -3.1 |
| Atrial fibrillation |  | 85 (9.5) | 1,212 (6.7) | 10.3 |  | 76 (9.1) | 83 (9.9) | -2.9 |
| Ischemic heart disease |  | 328 (36.8) | 6,711 (37.3) | -1.0 |  | 300 (35.9) | 313 (37.4) | -3.2 |
| Peripheral arterial disease |  | 45 (5.1) | 479 (2.7) | 12.4 |  | 44 (5.3) | 44 (5.3) | 0.0 |
| Hemorrhagic stroke |  | 16 (1.8) | 269 (1.5) | 2.4 |  | 16 (1.9) | 12 (1.4) | 3.7 |
| Ischemic stroke |  | 537 (60.3) | 7,740 (43.0) | 35.1 |  | 506 (60.5) | 511 (61.1) | -1.2 |
| Myocardial infarction |  | 381 (42.8) | 10,647 (59.2) | -33.2 |  | 353 (42.2) | 347 (41.5) | 1.5 |
| Chronic kidney disease |  | 90 (10.1) | 1,506 (8.4) | 6.0 |  | 82 (9.8) | 75 (9.0) | 2.9 |
| Severe liver impairment |  | X | 23 (0.1) | X |  | X | X | X |
| Hypothyroidism |  | 59 (6.6) | 1,017 (5.7) | 4.1 |  | 54 (6.5) | 52 (6.2) | 1.0 |
| Hyperthyroidism |  | 22 (2.5) | 255 (1.4) | 7.6 |  | 20 (2.4) | 20 (2.4) | 0.0 |
| Rheumatoid Arthritis |  | 19 (2.1) | 380 (2.1) | 0.1 |  | 18 (2.2) | 18 (2.2) | 0.0 |
| Osteoarthritis |  | 205 (23.0) | 3,454 (19.2) | 9.4 |  | 196 (23.4) | 204 (24.4) | -2.2 |
| Pre-existing muscle complaints |  | 98 (11.0) | 1,802 (10.0) | 3.2 |  | 91 (10.9) | 91 (10.9) | 0.0 |
| Musculoskeletal injuries |  | 285 (32.0) | 6,358 (35.3) | -7.1 |  | 266 (31.8) | 270 (32.3) | -1.0 |
| Chronic obstructive pulmonary disease |  | 53 (5.9) | 1,075 (6.0) | -0.1 |  | 48 (5.7) | 47 (5.6) | 0.5 |
| Macular degeneration |  | 8 (0.9) | 149 (0.8) | 0.8 |  | 7 (0.8) | 8 (1.0) | -1.3 |
| Falls^†^ |  | 42 (4.7) | 656 (3.6) | 5.3 |  | 38 (4.5) | 30 (3.6) | 4.8 |
| Pressure ulcer^†^ |  | X | 115 (0.6) | X |  | X | 7 (0.8) | X |
| Incontinence^†^ |  | 7 (0.8) | 209 (1.2) | -3.8 |  | 7 (0.8) | 7 (0.8) | 0.0 |
| Peripheral venous thrombosis^†^ |  | 19 (2.1) | 287 (1.6) | 4.0 |  | 15 (1.8) | 14 (1.7) | 0.9 |
| Pneumonia^†^ |  | 10 (1.1) | 194 (1.1) | 0.4 |  | 8 (1.0) | 6 (0.7) | 2.6 |
| Dysphagia^†^ |  | 9 (1.0) | 129 (0.7) | 3.2 |  | 6 (0.7) | 8 (1.0) | -2.6 |
| Anemia^†^ |  | 21 (2.4) | 466 (2.6) | -1.5 |  | 21 (2.5) | 19 (2.3) | 1.6 |
| Comedication, n (%) - in the 180 days before the cohort entry date | | | | |  |  |  |  |
| Fibrates |  | 25 (2.8) | 170 (0.9) | 13.8 |  | 22 (2.6) | 24 (2.9) | -1.5 |
| Amiodarone |  | 13 (1.5) | 275 (1.5) | -0.6 |  | 13 (1.6) | 15 (1.8) | -1.9 |
| Systemic corticosteroids |  | 40 (4.5) | 836 (4.6) | -0.7 |  | 37 (4.4) | 28 (3.3) | 5.6 |
| Antipsychotics |  | 8 (0.9) | 108 (0.6) | 3.5 |  | 6 (0.7) | 10 (1.2) | -4.9 |
| H_2_-receptor antagonists |  | 36 (4.0) | 944 (5.2) | -5.7 |  | 32 (3.8) | 26 (3.1) | 3.9 |
| Benzodiazepines |  | 95 (10.7) | 1,414 (7.9) | 9.7 |  | 92 (11.0) | 88 (10.5) | 1.5 |
| Number of cardiovascular drug classes |  |  |  |  |  |  |  |  |
| 0 |  | 68 (7.6) | 711 (4.0) | 15.8 |  | 63 (7.5) | 67 (8.0) | -1.8 |
| 1 to 3 |  | 560 (62.9) | 10,630 (59.1) | 7.8 |  | 525 (62.8) | 510 (61.0) | 3.7 |
| 4 to 10 |  | 263 (29.5) | 6,659 (37.0) | -15.9 |  | 248 (29.7) | 259 (31.0) | -2.9 |
| Number of general practitioner visits^‡^, mean (SD) |  | 22.4 (14.4) | 20.8 (15.0) | 10.3 |  | 22.1 (14.2) | 21.9 (14.2) | 1.3 |
| Hospitalization^†^, n (%) |  | 303 (34.0) | 9,760 (54.2) | -41.6 |  | 285 (34.1) | 275 (32.9) | 2.5 |
| Daily statin dose [mg], n (%) |  |  |  |  |  |  |  |  |
| 5 (rosuvastatin) vs 10 (atorvastatin) |  | 69 (7.7) | 5,264 (29.2) | -57.6 |  | 61 (7.3) | 64 (7.7) | -1.4 |
| 10 (rosuvastatin) vs 20 (atorvastatin) |  | 777 (87.2) | 2,990 (16.6) | 199.6 |  | 730 (87.3) | 727 (87.0) | 1.1 |
| 20 (rosuvastatin) vs 40 (atorvastatin) |  | 35 (3.9) | 3,903 (21.7) | -55.1 |  | 35 (4.2) | 35 (4.2) | 0.0 |
| 40 (rosuvastatin) vs 80 (atorvastatin) |  | 10 (1.1) | 5,843 (32.5) | -92.3 |  | 10 (1.2) | 10 (1.2) | 0.0 |
| Cohort entry date, n (%) |  |  |  |  |  |  |  |  |
| 2000-2001 |  | NA | NA |  |  | NA | NA |  |
| 2002-2003 |  | 211 (23.7) | 2,203 (12.2) | 30.1 |  | 195 (23.3) | 195 (23.3) | 0.0 |
| 2004-2005 |  | 389 (43.7) | 4,622 (25.7) | 38.5 |  | 384 (45.9) | 384 (45.9) | 0.0 |
| 2006-2007 |  | 126 (14.1) | 1,394 (7.7) | 20.6 |  | 121 (14.5) | 121 (14.5) | 0.0 |
| 2008-2009 |  | 63 (7.1) | 1,040 (5.8) | 5.3 |  | 53 (6.3) | 53 (6.3) | 0.0 |
| 2010-2011 |  | 42 (4.7) | 1,563 (8.7) | -15.9 |  | 32 (3.8) | 32 (3.8) | 0.0 |
| 2012-2013 |  | 27 (3.0) | 2,220 (12.3) | -35.5 |  | 22 (2.6) | 22 (2.6) | 0.0 |
| 2014-2015 |  | 14 (1.6) | 2,786 (15.5) | -51.4 |  | 14 (1.7) | 14 (1.7) | 0.0 |
| 2016-2017 |  | 19 (2.1) | 2,172 (12.1) | -39.4 |  | 15 (1.8) | 15 (1.8) | 0.0 |
| *SD* standard deviation; *X* cell contains <5 patients (not shown owing to ethics regulations to preserve confidentiality); *NA* not applicable  ^*^ Last record before the cohort entry date.  ^†^ Assessed in the 3 years before the cohort entry date.  ^‡^ Assessed in the 1 year before the cohort entry date. | | | | | | | | |

| **Appendix Table 7. Baseline covariates of users of simvastatin 40-80 mg and atorvastatin 10-20 mg (moderate- to high-intensity statin therapy) in the secondary prevention cohort before and after propensity score matching** | | | | | | | | |
| --- | --- | --- | --- | --- | --- | --- | --- | --- |
|  |  | **Cohort before**  **propensity score matching** | |  |  | **Cohort after**  **propensity score matching** | |  |
| **Covariate** |  | **Simvastatin**  **N = 28,142** | **Atorvastatin**  **N = 15,404** | **Absolute standardized difference (%)** |  | **Simvastatin**  **N = 6,716** | **Atorvastatin**  **N = 6,716** | **Absolute standardized difference (%)** |
| Age [years], mean (SD) |  | 64.8 (10.3) | 66.6 (9.3) | -18.1 |  | 66.9 (9.5) | 66.8 (9.5) | 0.8 |
| Male, n (%) |  | 17,169 (61.0) | 9,138 (59.3) | 3.4 |  | 4,064 (60.5) | 4,016 (59.8) | 1.5 |
| Current smoker^*^, n (%) |  | 7,267 (25.8) | 3,483 (22.6) | 7.5 |  | 1,571 (23.4) | 1,566 (23.3) | 0.2 |
| >14 units of alcohol per week^*^, n (%) |  | 2,854 (10.1) | 1,367 (8.9) | 4.3 |  | 615 (9.2) | 594 (8.8) | 1.1 |
| Obesity^*^, n (%) |  | 6,358 (22.6) | 3,422 (22.2) | 0.9 |  | 1,444 (21.5) | 1,418 (21.1) | 0.9 |
| Comorbidities, n (%) - at any time before the cohort entry date, if not specified otherwise | | | | |  |  |  |  |
| Hyperlipidemia |  | 11,229 (39.9) | 7,098 (46.1) | -12.5 |  | 2,886 (43.0) | 2,892 (43.1) | -0.2 |
| Diabetes mellitus |  | 2,354 (8.4) | 2,527 (16.4) | -24.6 |  | 1,004 (14.9) | 974 (14.5) | 1.3 |
| Hypertension |  | 11,331 (40.3) | 7,657 (49.7) | -19.1 |  | 3,338 (49.7) | 3,305 (49.2) | 1.0 |
| Heart failure |  | 1,135 (4.0) | 1,080 (7.0) | -13.1 |  | 443 (6.6) | 449 (6.7) | -0.4 |
| Atrial fibrillation |  | 2,185 (7.8) | 1,279 (8.3) | -2.0 |  | 577 (8.6) | 564 (8.4) | 0.7 |
| Ischemic heart disease |  | 7,229 (25.7) | 6,159 (40.0) | -30.8 |  | 2,407 (35.8) | 2,410 (35.9) | -0.1 |
| Peripheral arterial disease |  | 765 (2.7) | 861 (5.6) | -14.4 |  | 334 (5.0) | 331 (4.9) | 0.2 |
| Hemorrhagic stroke |  | 496 (1.8) | 283 (1.8) | -0.6 |  | 142 (2.1) | 135 (2.0) | 0.7 |
| Ischemic stroke |  | 17,304 (61.5) | 9,117 (59.2) | 4.7 |  | 4,116 (61.3) | 4,113 (61.2) | 0.1 |
| Myocardial infarction |  | 11,418 (40.6) | 6,932 (45.0) | -9.0 |  | 2,855 (42.5) | 2,862 (42.6) | -0.2 |
| Chronic kidney disease |  | 2,587 (9.2) | 1,332 (8.7) | 1.9 |  | 672 (10.0) | 689 (10.3) | -0.8 |
| Severe liver impairment |  | 60 (0.2) | 18 (0.1) | 2.4 |  | 13 (0.2) | 9 (0.1) | 1.5 |
| Hypothyroidism |  | 1,688 (6.0) | 978 (6.4) | -1.5 |  | 420 (6.3) | 411 (6.1) | 0.6 |
| Hyperthyroidism |  | 464 (1.7) | 279 (1.8) | -1.2 |  | 105 (1.6) | 118 (1.8) | -1.5 |
| Rheumatoid Arthritis |  | 602 (2.1) | 343 (2.2) | -0.6 |  | 159 (2.4) | 153 (2.3) | 0.6 |
| Osteoarthritis |  | 5,978 (21.2) | 3,335 (21.7) | -1.0 |  | 1,491 (22.2) | 1,505 (22.4) | -0.5 |
| Pre-existing muscle complaints |  | 2,788 (9.9) | 1,559 (10.1) | -0.7 |  | 695 (10.3) | 696 (10.4) | -0.0 |
| Musculoskeletal injuries |  | 9,576 (34.0) | 4,629 (30.1) | 8.5 |  | 2,081 (31.0) | 2,083 (31.0) | -0.1 |
| Chronic obstructive pulmonary disease |  | 1,918 (6.8) | 938 (6.1) | 3.0 |  | 464 (6.9) | 455 (6.8) | 0.5 |
| Macular degeneration |  | 286 (1.0) | 170 (1.1) | -0.9 |  | 79 (1.2) | 71 (1.1) | 1.1 |
| Falls^†^ |  | 1,332 (4.7) | 715 (4.6) | 0.4 |  | 345 (5.1) | 359 (5.3) | -0.9 |
| Pressure ulcer^†^ |  | 183 (0.7) | 127 (0.8) | -2.0 |  | 49 (0.7) | 49 (0.7) | 0.0 |
| Incontinence^†^ |  | 372 (1.3) | 202 (1.3) | 0.1 |  | 97 (1.4) | 103 (1.5) | -0.7 |
| Peripheral venous thrombosis^†^ |  | 430 (1.5) | 261 (1.7) | -1.3 |  | 115 (1.7) | 129 (1.9) | -1.6 |
| Pneumonia^†^ |  | 329 (1.2) | 160 (1.0) | 1.2 |  | 77 (1.1) | 78 (1.2) | -0.1 |
| Dysphagia^†^ |  | 269 (1.0) | 129 (0.8) | 1.3 |  | 56 (0.8) | 60 (0.9) | -0.6 |
| Anemia^†^ |  | 743 (2.6) | 449 (2.9) | -1.7 |  | 201 (3.0) | 208 (3.1) | -0.6 |
| Comedication, n (%) - in the 180 days before the cohort entry date | | | | |  |  |  |  |
| Fibrates |  | 169 (0.6) | 348 (2.3) | -14.0 |  | 81 (1.2) | 85 (1.3) | -0.5 |
| Amiodarone |  | 257 (0.9) | 334 (2.2) | -10.2 |  | 134 (2.0) | 134 (2.0) | 0.0 |
| Systemic corticosteroids |  | 1,388 (4.9) | 641 (4.2) | 3.7 |  | 300 (4.5) | 295 (4.4) | 0.4 |
| Antipsychotics |  | 194 (0.7) | 88 (0.6) | 1.5 |  | 43 (0.6) | 45 (0.7) | -0.4 |
| H_2_-receptor antagonists |  | 1,118 (4.0) | 899 (5.8) | -8.6 |  | 324 (4.8) | 306 (4.6) | 1.3 |
| Benzodiazepines |  | 2,295 (8.2) | 1,476 (9.6) | -5.0 |  | 663 (9.9) | 636 (9.5) | 1.4 |
| Number of cardiovascular drug classes |  |  |  |  |  |  |  |  |
| 0 |  | 1,120 (4.0) | 884 (5.7) | -8.2 |  | 330 (4.9) | 347 (5.2) | -1.2 |
| 1 to 3 |  | 18,917 (67.2) | 9,763 (63.4) | 8.1 |  | 4,223 (62.9) | 4,209 (62.7) | 0.4 |
| 4 to 10 |  | 8,105 (28.8) | 4,757 (30.9) | -4.5 |  | 2,163 (32.2) | 2,160 (32.2) | 0.1 |
| Number of general practitioner visits^‡^, mean (SD) |  | 21.0 (14.6) | 21.5 (14.0) | -3.5 |  | 21.8 (14.4) | 21.9 (14.0) | -0.4 |
| Hospitalization^†^, n (%) |  | 14,699 (52.2) | 5,376 (34.9) | 35.5 |  | 2,439 (36.3) | 2,453 (36.5) | -0.4 |
| Daily statin dose [mg], n (%) |  |  |  |  |  |  |  |  |
| 40 (simvastatin) vs 10 (atorvastatin) |  | 3,917 (25.4) | 11,487 (74.6) | 80.5 |  | 6,613 (98.5) | 6,614 (98.5) | -0.1 |
| 80 (simvastatin) vs 20 (atorvastatin) |  | 110 (0.4) | 28,032 (99.6) | -80.5 |  | 103 (1.5) | 102 (1.5) | 0.1 |
| Cohort entry date, n (%) |  |  |  |  |  |  |  |  |
| 2000-2001 |  | 229 (0.8) | 4,158 (27.0) | -81.7 |  | 229 (3.4) | 229 (3.4) | 0.0 |
| 2002-2003 |  | 3,216 (11.4) | 5,080 (33.0) | -53.7 |  | 2,877 (42.8) | 2,877 (42.8) | 0.0 |
| 2004-2005 |  | 5,058 (18.0) | 3,811 (24.7) | -16.6 |  | 2,630 (39.2) | 2,630 (39.2) | 0.0 |
| 2006-2007 |  | 5,479 (19.5) | 625 (4.1) | 49.3 |  | 342 (5.1) | 342 (5.1) | 0.0 |
| 2008-2009 |  | 5,307 (18.9) | 152 (1.0) | 62.6 |  | 101 (1.5) | 101 (1.5) | 0.0 |
| 2010-2011 |  | 4,278 (15.2) | 114 (0.7) | 55.4 |  | 71 (1.1) | 71 (1.1) | 0.0 |
| 2012-2013 |  | 3,027 (10.8) | 402 (2.6) | 33.1 |  | 189 (2.8) | 189 (2.8) | 0.0 |
| 2014-2015 |  | 1,250 (4.4) | 656 (4.3) | 0.9 |  | 198 (2.9) | 198 (2.9) | 0.0 |
| 2016-2017 |  | 298 (1.1) | 406 (2.6) | -11.7 |  | 79 (1.2) | 79 (1.2) | 0.0 |
| *SD* standard deviation  ^*^ Last record before the cohort entry date.  ^†^ Assessed in the 3 years before the cohort entry date.  ^‡^ Assessed in the 1 year before the cohort entry date. | | | | | | | | |

| **Appendix Table 8. Censoring reasons and duration of follow-up for the secondary prevention cohorts before and after propensity score matching** | | | | | | | | | |  |
| --- | --- | --- | --- | --- | --- | --- | --- | --- | --- | --- |
|  |  | **Low-intensity statin therapy** | |  | **Moderate- to high-intensity statin therapy** | | | |  | |
|  |  | **Pravastatin 20-40 mg** | **Simvastatin**  **10-20 mg** |  | **Rosuvastatin**  **5-40 mg** | **Atorvastatin**  **10-80 mg** |  | **Simvastatin**  **40-80 mg** | **Atorvastatin**  **10-20 mg** | |
| **Before propensity score matching** |  | **N = 4,139** | **N = 24,836** |  | **N = 891** | **N = 18,000** |  | **N = 28,142** | **N = 15,404** | |
| **Censoring reasons, n (%)** |  |  |  |  |  |  |  |  |  | |
| Muscular event (Outcome) |  | 43 (1.0) | 276 (1.1) |  | 14 (1.6) | 259 (1.4) |  | 467 (1.7) | 145 (0.9) | |
| Recording of statin intolerance |  | 7 (0.2) | 187 (0.8) |  | 5 (0.6) | 80 (0.4) |  | 206 (0.7) | 60 (0.4) | |
| Treatment switch |  | 547 (13.2) | 1,732 (7.0) |  | 73 (8.2) | 1,345 (7.5) |  | 2,744 (9.8) | 1,098 (7.1) | |
| Discontinuation of statin treatment |  | 426 (10.3) | 3,928 (15.8) |  | 127 (14.3) | 1,688 (9.4) |  | 3,016 (10.7) | 2,182 (14.2) | |
| Death |  | 40 (1.0) | 191 (0.8) |  | 5 (0.6) | 134 (0.7) |  | 239 (0.8) | 114 (0.7) | |
| Recording of an exclusion criterion |  | 90 (2.2) | 586 (2.4) |  | 28 (3.1) | 384 (2.1) |  | 756 (2.7) | 311 (2.0) | |
| End of study enrollment |  | 2,986 (72.1) | 17,936 (72.2) |  | 639 (71.7) | 14,110 (78.4) |  | 20,714 (73.6) | 11,494 (74.6) | |
| **Duration of follow-up** |  |  |  |  |  |  |  |  |  | |
| Mean number of days (standard deviation) |  | 302.9 (105.8) | 301.8 (105.9) |  | 299.9 (107.0) | 297.2 (109.7) |  | 300.2 (108.9) | 304.8 (104.1) | |
| Median number of days (interquartile range) |  | 365 (259-365) | 365 (251-365) |  | 365 (236-365) | 365 (237-365) |  | 365 (250-365) | 365 (266-365) | |
| **After propensity score matching** |  | **N = 4,121** | **N = 4,121** |  | **N = 836** | **N = 836** |  | **N = 6,716** | **N = 6,716** | |
| **Censoring reasons, n (%)** |  |  |  |  |  |  |  |  |  | |
| Muscular event (Outcome) |  | 43 (1.0) | 42 (1.0) |  | 13 (1.6) | 14 (1.7) |  | 95 (1.4) | 66 (1.0) | |
| Recording of statin intolerance |  | 7 (0.2) | 15 (0.4) |  | 5 (0.6) | X |  | 47 (0.7) | 43 (0.6) | |
| Treatment switch |  | 546 (13.2) | 286 (6.9) |  | 69 (8.3) | 65 (7.8) |  | 528 (7.9) | 580 (8.6) | |
| Discontinuation of statin treatment |  | 423 (10.3) | 556 (13.5) |  | 120 (14.4) | 116 (13.9) |  | 750 (11.2) | 1,007 (15.0) | |
| Death |  | 40 (1.0) | 37 (0.9) |  | X | 5 (0.6) |  | 83 (1.2) | 58 (0.9) | |
| Recording of an exclusion criterion |  | 89 (2.2) | 92 (2.2) |  | 24 (2.9) | 11 (1.3) |  | 188 (2.8) | 158 (2.4) | |
| End of study enrollment |  | 2,973 (72.1) | 3,093 (75.1) |  | 601 (71.9) | 623 (74.5) |  | 5,025 (74.8) | 4,804 (71.5) | |
| **Duration of follow-up** |  |  |  |  |  |  |  |  |  | |
| Mean number of days (standard deviation) |  | 303.0 (105.8) | 307.7 (102.6) |  | 299.8 (107.3) | 304.8 (105.8) |  | 306.1 (104.0) | 302.0 (105.1) | |
| Median number of days (interquartile range) |  | 365 (260-365) | 365 (286-365) |  | 365 (237-365) | 365 (271-365) |  | 365 (278-365) | 365 (254-365) | |
| *X* cell contains <5 patients (not shown owing to ethics regulations to preserve confidentiality) | | | | | | | | | |  |

| **Appendix Table 9. Baseline covariates by treatment group in the primary prevention cohorts of pravastatin vs simvastatin and rosuvastatin vs atorvastatin before propensity score matching (simvastatin vs atorvastatin in Appendix Table 4)** | | | | | | |
| --- | --- | --- | --- | --- | --- | --- |
|  | **Low-intensity statin therapy** | |  | **Moderate- to high-intensity statin therapy** | |  |
| **Covariate** | **Pravastatin**  **N = 9,710** | **Simvastatin**  **N = 179,416** | **ASD (%)** | **Rosuvastatin**  **N = 7,057** | **Atorvastatin**  **N = 85,699** | **ASD (%)** |
| Age [years], mean (SD) | 63.1 (9.8) | 62.5 (9.7) | 6.1 | 61.0 (9.9) | 61.4 (9.9) | -4.3 |
| Male, n (%) | 4,891 (50.4) | 86,857 (48.4) | 3.9 | 3,427 (48.6) | 42,933 (50.1) | -3.1 |
| Current smoker^*^, n (%) | 1,950 (20.1) | 34,159 (19.0) | 2.6 | 1,381 (19.6) | 16,019 (18.7) | 2.2 |
| >14 alcohol units/week^*^, n (%) | 809 (8.3) | 17,594 (9.8) | -5.1 | 710 (10.1) | 8,899 (10.4) | -1.1 |
| Obesity^*^, n (%) | 2,533 (26.1) | 53,936 (30.1) | -8.9 | 2,111 (29.9) | 28,427 (33.2) | -7.0 |
| Comorbidities, n (%) - at any time before the cohort entry date, if not specified otherwise | | | | | |  |
| Hyperlipidemia | 4,916 (50.6) | 123,363 (68.8) | -37.6 | 4,894 (69.3) | 61,575 (71.9) | -5.5 |
| Diabetes mellitus | 2,099 (21.6) | 38,844 (21.7) | -0.1 | 1,507 (21.4) | 19,503 (22.8) | -3.4 |
| Hypertension | 4,751 (48.9) | 93,197 (51.9) | -6.0 | 3,626 (51.4) | 41,032 (47.9) | 7.0 |
| Heart failure | 401 (4.1) | 2,879 (1.6) | 15.2 | 116 (1.6) | 1,406 (1.6) | 0.0 |
| Atrial fibrillation | 793 (8.2) | 6,101 (3.4) | 20.5 | 217 (3.1) | 3,466 (4.0) | -5.2 |
| Ischemic heart disease | 2,629 (27.1) | 18,479 (10.3) | 44.1 | 700 (9.9) | 8,602 (10.0) | -0.4 |
| Peripheral arterial disease | 492 (5.1) | 4,541 (2.5) | 13.3 | 148 (2.1) | 1,788 (2.1) | 0.1 |
| Hemorrhagic stroke | 83 (0.9) | 1,029 (0.6) | 3.3 | 31 (0.4) | 550 (0.6) | -2.8 |
| Chronic kidney disease | 578 (6.0) | 15,871 (8.8) | -11.1 | 483 (6.8) | 6,617 (7.7) | -3.4 |
| Severe liver impairment | 30 (0.3) | 230 (0.1) | 3.9 | 6 (0.1) | 136 (0.2) | -2.1 |
| Hypothyroidism | 658 (6.8) | 13,649 (7.6) | -3.2 | 522 (7.4) | 6,545 (7.6) | -0.9 |
| Hyperthyroidism | 161 (1.7) | 3,042 (1.7) | -0.3 | 104 (1.5) | 1,460 (1.7) | -1.8 |
| Rheumatoid Arthritis | 166 (1.7) | 2,742 (1.5) | 1.4 | 100 (1.4) | 1,459 (1.7) | -2.3 |
| Osteoarthritis | 1,863 (19.2) | 34,841 (19.4) | -0.6 | 1,251 (17.7) | 16,424 (19.2) | -3.7 |
| Pre-existing muscle  complaints | 844 (8.7) | 16,034 (8.9) | -0.9 | 576 (8.2) | 8,716 (10.2) | -7.0 |
| Musculoskeletal injuries | 2,823 (29.1) | 56,962 (31.7) | -5.8 | 2,150 (30.5) | 29,770 (34.7) | -9.1 |
| COPD | 439 (4.5) | 7,030 (3.9) | 3.0 | 231 (3.3) | 3,563 (4.2) | -4.7 |
| Macular degeneration | 84 (0.9) | 1,381(0.8) | 1.1 | 37 (0.5) | 577 (0.7) | -1.9 |
| Falls^†^ | 266 (2.7) | 5,462 (3.0) | -1.8 | 175 (2.5) | 2,476 (2.9) | -2.5 |
| Pressure ulcer^†^ | 54 (0.6) | 723 (0.4) | 2.2 | 26 (0.4) | 335 (0.4) | -0.4 |
| Incontinence^†^ | 70 (0.7) | 1,645 (0.9) | -2.2 | 53 (0.8) | 849 (1.0) | -2.6 |
| Peripheral venous  thrombosis^†^ | 155 (1.6) | 2,297 (1.3) | 2.7 | 98 (1.4) | 1,069 (1.2) | 1.2 |
| Pneumonia^†^ | 66 (0.7) | 868 (0.5) | 2.6 | 31 (0.4) | 480 (0.6) | -1.7 |
| Dysphagia^†^ | 65 (0.7) | 1,078 (0.6) | 0.9 | 33 (0.5) | 486 (0.6) | -1.4 |
| Anemia^†^ | 207 (2.1) | 3,485 (1.9) | 1.3 | 94 (1.3) | 1,604 (1.9) | -4.3 |
| Comedication, n (%) - in the 180 days before the cohort entry date | | | |  |  |  |
| Fibrates | 132 (1.4) | 1,243 (0.7) | 6.6 | 180 (2.6) | 818 (1.0) | 12.2 |
| Amiodarone | 185 (1.9) | 930 (0.5) | 12.7 | 21 (0.3) | 466 (0.5) | -3.8 |
| Systemic corticosteroids | 384 (4.0) | 5,636 (3.1) | 4.4 | 218 (3.1) | 3,385 (3.9) | -4.7 |
| Antipsychotics | 41 (0.4) | 1,135 (0.6) | -2.9 | 47 (0.7) | 559 (0.7) | 0.2 |
| H_2_-receptor antagonists | 539 (5.6) | 5,731 (3.2) | 11.5 | 199 (2.8) | 2,579 (3.0) | -1.1 |
| Benzodiazepines | 844 (8.7) | 11,841 (6.6) | 7.9 | 530 (7.5) | 5,630 (6.6) | 3.7 |
| Number of cardiovascular  drug classes |  |  |  |  |  |  |
| 0 | 1,760 (18.1) | 52,934 (29.5) | -27.0 | 2,248 (31.9) | 27,468 (32.1) | -0.4 |
| 1 to 3 | 5,810 (59.8) | 108,015 (60.2) | -0.8 | 4,077 (57.8) | 49,323 (57.6) | 0.4 |
| 4 to 10 | 2,140 (22.0) | 18,467 (10.3) | 32.3 | 732 (10.4) | 8,908 (10.4) | -0.1 |
| Number of general practitioner visits^‡^, mean (SD) | 20.6 (13.5) | 19.6 (12.3) | 7.6 | 19.1 (12.2) | 20.8 (13.5) | -13.3 |
| Hospitalization^†^, n (%) | 2,748 (28.3) | 47,739 (26.6) | 3.8 | 1,735 (24.6) | 29,538 (34.5) | -21.8 |
| Daily statin dose [mg], n (%) |  |  |  |  |  |  |
| 20 (P), 10 (S) | 4,412 (45.4) | 50,581 (28.2) | 36.3 | NA | NA |  |
| 40 (P), 20 (S) | 5,298 (54.6) | 128,835 (71.8) | -36.3 | NA | NA |  |
| 40 (S), 5 (R), 10 (A) | NA | NA |  | 696 (9.9) | 42,765 (49.9) | -97.3 |
| 80 (S), 10 (R), 20 (A) | NA | NA |  | 6,112 (86.6) | 32,558 (38.0) | 116.0 |
| 20 (R), 40 (A) | NA | NA |  | 226 (3.2) | 8,440 (9.8) | -27.2 |
| 40 (R), 80 (A) | NA | NA |  | 23 (0.3) | 1,936 (2.3) | -17.2 |
| Cohort entry date, n (%) |  |  |  |  |  |  |
| 2000-2001 | 2,912 (30.0) | 14,676 (8.2) | 57.8 | NA | NA |  |
| 2002-2003 | 4,202 (43.3) | 25,378 (14.1) | 68.0 | 1,425 (20.2) | 11,078 (12.9) | 19.6 |
| 2004-2005 | 1,409 (14.5) | 39,146 (21.8) | -19.0 | 3,132 (44.4) | 25,631 (29.9) | 30.3 |
| 2006-2007 | 322 (3.3) | 43,099 (24.0) | -63.2 | 1,263 (17.9) | 6,664 (7.8) | 30.6 |
| 2008-2009 | 216 (2.2) | 25,547 (14.2) | -44.8 | 557 (7.9) | 1,994 (2.3) | 25.5 |
| 2010-2011 | 246 (2.5) | 13,840 (7.7) | -23.7 | 344 (4.9) | 1,560 (1.8) | 17.0 |
| 2012-2013 | 284 (2.9) | 11,139 (6.2) | -15.8 | 147 (2.1) | 7,916 (9.2) | -31.3 |
| 2014-2015 | 89 (0.9) | 5,010 (2.8) | -13.9 | 110 (1.6) | 16,239 (18.9) | -59.8 |
| 2016-2017 | 30 (0.3) | 1,581 (0.9) | -7.4 | 79 (1.1) | 14,617 (17.1) | -57.7 |
| *ASD* absolute standardized difference; *SD* standard deviation; *COPD* chronic obstructive pulmonary disease; *P* pravastatin; *S* simvastatin; *R* rosuvastatin; *A* atorvastatin; *NA* not applicable  ^*^ Last record before the cohort entry date.  ^†^ Assessed in the 3 years before the cohort entry date.  ^‡^ Assessed in the 1 year before the cohort entry date. | | | | | | |

| **Appendix Table 10. Censoring reasons and duration of follow-up for the primary prevention cohorts before propensity score matching** | | | | | | | | | |  |
| --- | --- | --- | --- | --- | --- | --- | --- | --- | --- | --- |
|  |  | **Low-intensity statin therapy** | |  | **Moderate- to high-intensity statin therapy** | | | |  | |
|  |  | **Pravastatin 20-40 mg**  **N = 9,710** | **Simvastatin**  **10-20 mg**  **N = 179,416** |  | **Rosuvastatin**  **5-40 mg**  **N = 7,057** | **Atorvastatin**  **10-80 mg**  **N = 85,699** |  | **Simvastatin**  **40-80 mg**  **N = 161,572** | **Atorvastatin**  **10-20 mg**  **N = 101,359** | |
| **Censoring reasons, n (%)** |  |  |  |  |  |  |  |  |  | |
| Muscular event (Outcome) |  | 82 (0.8) | 2,205 (1.2) |  | 101 (1.4) | 854 (1.0) |  | 2,456 (1.5) | 957 (0.9) | |
| Recording of statin intolerance |  | 17 (0.2) | 1,254 (0.7) |  | 20 (0.3) | 340 (0.4) |  | 1,155 (0.7) | 313 (0.3) | |
| Treatment switch |  | 1,287 (13.3) | 12,302 (6.9) |  | 682 (9.7) | 5,995 (7.0) |  | 14,222 (8.8) | 6,828 (6.7) | |
| Discontinuation of statin treatment |  | 1,760 (18.1) | 38,286 (21.3) |  | 1,414 (20.0) | 16,801 (19.6) |  | 35,060 (21.7) | 19,802 (19.5) | |
| Death |  | 40 (0.4) | 385 (0.2) |  | 8 (0.1) | 179 (0.2) |  | 416 (0.3) | 199 (0.2) | |
| Recording of an exclusion criterion |  | 198 (2.0) | 3,022 (1.7) |  | 126 (1.8) | 1,465 (1.7) |  | 3,002 (1.9) | 1,696 (1.7) | |
| Myocardial infarction or ischemic stroke |  | 283 (2.9) | 2,156 (1.2) |  | 59 (0.8) | 1,152 (1.3) |  | 2,429 (1.5) | 1,278 (1.3) | |
| End of study enrollment |  | 6,043 (62.2) | 119,806 (66.8) |  | 4,647 (65.8) | 58,913 (68.7) |  | 102,832 (63.6) | 70,286 (69.3) | |
| **Duration of follow-up** |  |  |  |  |  |  |  |  |  | |
| Mean number of days (standard deviation) |  | 285.1 (112.4) | 291.4 (109.7) |  | 292.1 (110.4) | 274.2 (117.0) |  | 281.4 (114.9) | 283.4 (113.2) | |
| Median number of days (interquartile range) |  | 365 (184-365) | 365 (202-365) |  | 365 (205-365) | 365 (155-365) |  | 365 (174-365) | 365 (176-365) | |

| **Appendix Table 11. Hazard ratios for muscular events in the primary prevention cohorts before propensity score matching, using multivariable logistic regression models** | | | | | | | | |
| --- | --- | --- | --- | --- | --- | --- | --- | --- |
|  | **Number of events** | |  | **Total person-years of follow-up** | |  | **HR (95% CI)** |  |
|  | **Exposed** | **Comparator** |  | **Exposed** | **Comparator** |  | **Crude** | **Adjusted** |
| **Low-intensity statin therapy** |  |  |  |  |  |  |  |  |
| **Pravastatin vs Simvastatin (ref)** |  |  |  |  |  |  |  |  |
| **Overall** | 82 (0.8) | 2,205 (1.2) |  | 7,584 | 143,220 |  | 0.70 (0.56-0.87) | 0.87 (0.69-1.09) |
| **Subgroup analyses** |  |  |  |  |  |  |  |  |
| Male | 33 | 955 |  | 3,868 | 69,725 |  | 0.62 (0.44-0.88) | 0.75 (0.53-1.08) |
| Female | 49 | 1,250 |  | 3,716 | 73,496 |  | 0.77 (0.58-1.02) | 0.96 (0.71-1.29) |
| 40-64 years | 39 | 1,211 |  | 3,913 | 78,433 |  | 0.64 (0.47-0.88) | 0.76 (0.55-1.06) |
| ≥65 years | 43 | 994 |  | 3,671 | 64,787 |  | 0.76 (0.56-1.03) | 0.98 (0.71-1.34) |
| 20 vs 10 mg | 35 | 567 |  | 3,470 | 40,673 |  | 0.72 (0.51-1.01) | 0.79 (0.56-1.12) |
| 40 vs 20 mg | 47 | 1,638 |  | 4,114 | 102,547 |  | 0.71 (0.53-0.95) | 0.95 (0.70-1.29) |
| **Sensitivity analyses** |  |  |  |  |  |  |  |  |
| No muscle complaints before CED | 71 | 1,833 |  | 6,937 | 130,826 |  | 0.73 (0.57-0.92) | 0.93 (0.72-1.19) |
| No use of CYP3A4 inhibiting drugs^*^ | 57 | 1,664 |  | 5,277 | 106,909 |  | 0.69 (0.53-0.90) | 0.86 (0.65-1.13) |
| **Additional analyses** |  |  |  |  |  |  |  |  |
| Censoring if dosage change | 75 | 1,948 |  | 7,040 | 127,017 |  | 0.70 (0.56-0.88) | 0.86 (0.68-1.09) |
| Broader outcome definition^†^ | 99 | 3,459 |  | 7,584 | 143,220 |  | 0.54 (0.44-0.66) | 0.74 (0.60-0.91) |
| **Moderate- to high-intensity statin therapy** | |  |  |  |  |  |  |  |
| **Rosuvastatin vs Atorvastatin (ref)** |  |  |  |  |  |  |  |  |
| **Overall** | 101 (1.4) | 854 (1.0) |  | 5,648 | 64,369 |  | 1.36 (1.11-1.68) | 1.28 (1.02-1.61) |
| **Subgroup analyses** |  |  |  |  |  |  |  |  |
| Male | 42 | 357 |  | 2,776 | 32,408 |  | 1.39 (1.01-1.91) | 1.22 (0.86-1.75) |
| Female | 59 | 497 |  | 2,872 | 31,961 |  | 1.34 (1.02-1.75) | 1.32 (0.97-1.78) |
| 40-64 years | 59 | 496 |  | 3,475 | 38,095 |  | 1.32 (1.01-1.73) | 1.19 (0.88-1.60) |
| ≥65 years | 42 | 358 |  | 2,173 | 26,274 |  | 1.43 (1.04-1.97) | 1.44 (1.00-2.07) |
| 5-10 vs 10-20 mg | 97 | 725 |  | 5,452 | 56,895 |  | 1.41 (1.14-1.75) | 1.28 (1.01-1.63) |
| 20-40 vs 40-80 mg | X | 129 |  | 196 | 7,474 |  | 1.20 (0.44-3.25) | 1.07 (0.39-2.95) |
| **Sensitivity analyses** |  |  |  |  |  |  |  |  |
| No muscle complaints before CED | 88 | 692 |  | 5,198 | 58,043 |  | 1.44 (1.15-1.79) | 1.35 (1.05-1.73) |
| No use of CYP3A4 inhibiting drugs^*^ | 79 | 638 |  | 4,477 | 47,122 |  | 1.32 (1.04-1.67) | 1.29 (0.99-1.67) |
| **Additional analyses** |  |  |  |  |  |  |  |  |
| Censoring if dosage change | 97 | 803 |  | 5,412 | 59,768 |  | 1.36 (1.10-1.68) | 1.25 (0.98-1.58) |
| Broader outcome definition^†^ | 121 | 1,194 |  | 5,648 | 64,369 |  | 1.17 (0.97-1.41) | 1.12 (0.92-1.38) |
| **Simvastatin vs Atorvastatin (ref)** |  |  |  |  |  |  |  |  |
| **Overall** | 2,456 (1.5) | 957 (0.9) |  | 124,546 | 78,697 |  | 1.62 (1.50-1.75) | 1.37 (1.23-1.53) |
| **Subgroup analyses** |  |  |  |  |  |  |  |  |
| Male | 1,187 | 397 |  | 68,659 | 38,564 |  | 1.68 (1.50-1.88) | 1.41 (1.19-1.67) |
| Female | 1,269 | 560 |  | 55,888 | 40,133 |  | 1.63 (1.47-1.80) | 1.35 (1.16-1.56) |
| 40-64 years | 1,400 | 536 |  | 76,666 | 45,063 |  | 1.53 (1.39-1.69) | 1.27 (1.09-1.47) |
| ≥65 years | 1,056 | 421 |  | 47,880 | 33,634 |  | 1.77 (1.58-1.98) | 1.50 (1.27-1.77) |
| 40 vs 10 mg | 2,453 | 635 |  | 124,347 | 52,414 |  | 1.62 (1.48-1.76) | 1.37 (1.22-1.54) |
| 80 vs 20 mg | X | 322 |  | 200 | 26,283 |  | 1.24 (0.40-3.88) | 1.28 (0.41-4.05) |
| **Sensitivity analyses** |  |  |  |  |  |  |  |  |
| No muscle complaints before CED | 2,019 | 779 |  | 112,531 | 71,549 |  | 1.65 (1.52-1.79) | 1.35 (1.20-1.53) |
| No use of CYP3A4 inhibiting drugs^*^ | 1,839 | 715 |  | 93,640 | 57,881 |  | 1.59 (1.46-1.73) | 1.29 (1.13-1.46) |
| **Additional analyses** |  |  |  |  |  |  |  |  |
| Censoring if dosage change | 2,388 | 894 |  | 119,819 | 73,121 |  | 1.64 (1.52-1.77) | 1.40 (1.24-1.57) |
| Broader outcome definition^†^ | 3,611 | 1,270 |  | 124,546 | 78,697 |  | 1.79 (1.68-1.91) | 1.38 (1.25-1.51) |
| *HR* hazard ratio; *CI* confidence interval; *Ref* reference; *CYP3A4* Cytochrome P450 3A4; *X* cell contains <5 patients (not shown owing to ethics regulations to preserve confidentiality)  ^*^ The analysis was restricted to patients with no prescription for azole antifungals, macrolide antibiotics, cimetidine, cyclosporine, nefazodone, amiodarone, amlodipine, diltiazem, and verapamil within 180 days before the cohort entry date. We censored patients on the day of a first prescription for one of the drugs during follow-up.  ^†^ Any recorded Read code for ‘statin intolerance’ qualified as an outcome of interest. | | | | | | | | |

| **Appendix Table 12. Hazard ratios for muscular events in the secondary prevention cohorts before propensity score matching, using multivariable logistic regression models, and after propensity score matching** | | | | | | | | | | | |
| --- | --- | --- | --- | --- | --- | --- | --- | --- | --- | --- | --- |
|  | **Cohort before propensity score matching** | | | |  |  | **Cohort after propensity score matching** | | | |  |
|  | **Number of events** | | **Total person-years of follow-up** | | **HR (95% CI)** |  | **Number of events** | | **Total person-years of follow-up** | | **HR (95% CI)** |
|  | **Exposed** | **Comparator** | **Exposed** | **Comparator** | **Crude** | **Adjusted** | **Exposed** | **Comparator** | **Exposed** | **Comparator** |  |
| **Low-intensity statin therapy** | | |  |  |  |  |  |  |  |  |  |
| **Pravastatin vs Simvastatin (ref)** | | |  |  |  |  |  |  |  |  |  |
| **Overall** | 43 | 276 | 3,435 | 20,535 | 0.93 (0.68-1.29) | 1.09 (0.78-1.52) | 43 | 42 | 3,420 | 3,474 | 1.04 (0.68-1.59) |
| **Additional analysis** | | |  |  |  |  |  |  |  |  |  |
| Broader outcome definition^*^ | | |  |  |  |  |  |  |  |  |  |
|  | 50 | 463 | 3,435 | 20,535 | 0.65 (0.48-0.87) | 0.94 (0.69-1.26) | 50 | 57 | 3,420 | 3,474 | 0.89 (0.61-1.30) |
| **Moderate- to high-intensity statin therapy** | | | |  |  |  |  |  |  |  |  |
| **Rosuvastatin vs Atorvastatin (ref)** | | | |  |  |  |  |  |  |  |  |
| **Overall** | 14 | 259 | 732 | 14,658 | 1.08 (0.63-1.85) | 1.23 (0.68-2.23) | 13 | 14 | 687 | 698 | 0.93 (0.44-1.99) |
| **Additional analysis** | |  |  |  |  |  |  |  |  |  |  |
| Broader outcome definition^*^ | | |  |  |  |  |  |  |  |  |  |
|  | 19 | 339 | 732 | 14,658 | 1.12 (0.71-1.78) | 1.19 (0.71-1.98) | 18 | 16 | 687 | 698 | 1.13 (0.58-2.22) |
| **Simvastatin vs Atorvastatin (ref)** | | |  |  |  |  |  |  |  |  |  |
| **Overall** | 467 | 145 | 23,145 | 12,864 | 1.79 (1.48-2.15) | 1.39 (1.07-1.80) | 95 | 66 | 5,633 | 5,557 | 1.43 (1.04-1.95) |
| **Additional analysis** | | |  |  |  |  |  |  |  |  |  |
| Broader outcome definition^*^ | | |  |  |  |  |  |  |  |  |  |
|  | 673 | 205 | 23,145 | 12,864 | 1.82 (1.56-2.13) | 1.31 (1.06-1.62) | 142 | 109 | 5,633 | 5’557 | 1.29 (1.01-1.66) |
| *HR* hazard ratio; *CI* confidence interval; *Ref* reference  ^*^ Any recorded Read code for ‘statin intolerance’ qualified as an outcome of interest. | | | | | | | | | | | |

**Appendix Figure 1.** **Flow chart of the selection of the study population**

**Cohort III** (before PS matching): simvastatin 40-80 mg vs atorvastatin 10-20 mg

**N = 161,572 vs 101,359**

First prescription for fluvastatin, N=7,829

First prescription for cerivastatin, N=6,601

First prescription for pravastatin 10 mg, N=10,316

No statin prescription, N=9,265,941

Prevalent statin use, N=102,776

Initiation of >1 type of statin on same day, N=508

Aged <40 or >80 years, N=149,473

Clinical Practice Research Datalink (CPRD) GOLD - January 2000 to December 2017

**N = 10,507,304**

Other diagnosis leading to study exclusion, N=143,943

Record of rhabdomyolysis, myositis, myopathy, or prior statin intolerance, N=1,748

<3 years of medical records, N=264,991

Patients aged 40 to 80 years with a first statin prescription

**N = 988,606**

First statin prescription for pravastatin 20-40 mg, rosuvastatin 5-40 mg, atorvastatin 10-80 mg, or simvastatin 10-80 mg

**N = 963,860**

≥ 3 years of medical records in CPRD GOLD before first statin prescription

**N = 698,869**

No record of rhabdomyolysis, myositis, or myopathy before first statin prescription

**N = 697,121**

No diagnosis of a primary muscle disorder, myoneural disorder, a disorder associated with muscle pain, cancer, alcoholism or other substance abuse, or HIV before first statin prescription

**N = 553,178**

Primary prevention

of cardiovascular disease

**N = 469,860**

**Cohort I** (before PS matching): pravastatin 20-40 mg vs simvastatin 10-20 mg

**N = 9,710 vs 179,416**

**Cohort II** (before PS matching): rosuvastatin 5-40 mg vs atorvastatin 10-80 mg

**N = 7,057 vs 85,699**

(patients with a first prescription in March 2003 or thereafter)

Secondary prevention

of cardiovascular disease

**N = 83,318**

**Cohort VI** (before PS matching): simvastatin 40-80 mg vs atorvastatin 10-20 mg

**N = 28,142 vs 15,404**

**Cohort IV** (before PS matching): pravastatin 20-40 mg vs simvastatin 10-20 mg

**N = 4,139 vs 24,836**

**Cohort V** (before PS matching): rosuvastatin 5-40 mg vs atorvastatin 10-80 mg

**N = 891 vs 18,000**

(patients with a first prescription in March 2003 or thereafter)

**Appendix Figure 2. Kaplan Meier curves for muscular event-free survival after statin initiation in the primary prevention cohorts after propensity score matching**

**Appendix Figure 3. Hazard ratios for muscular events over time in the primary prevention cohorts after propensity score matching**


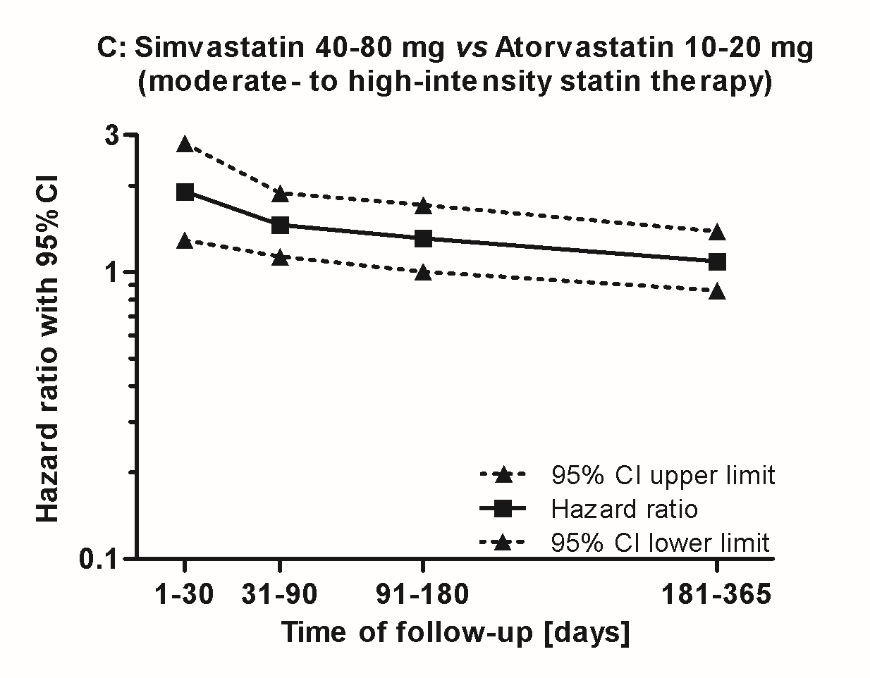

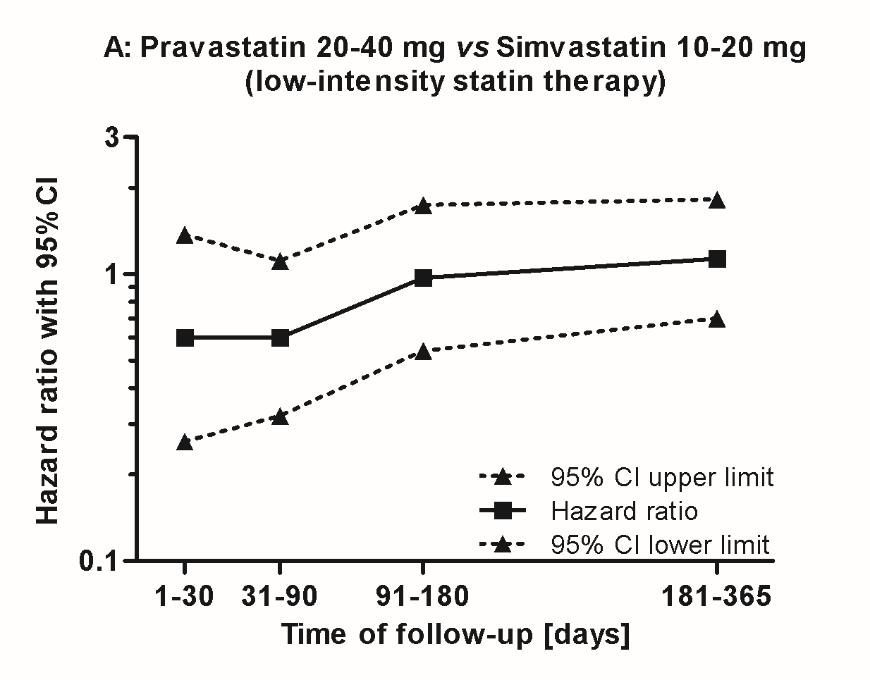

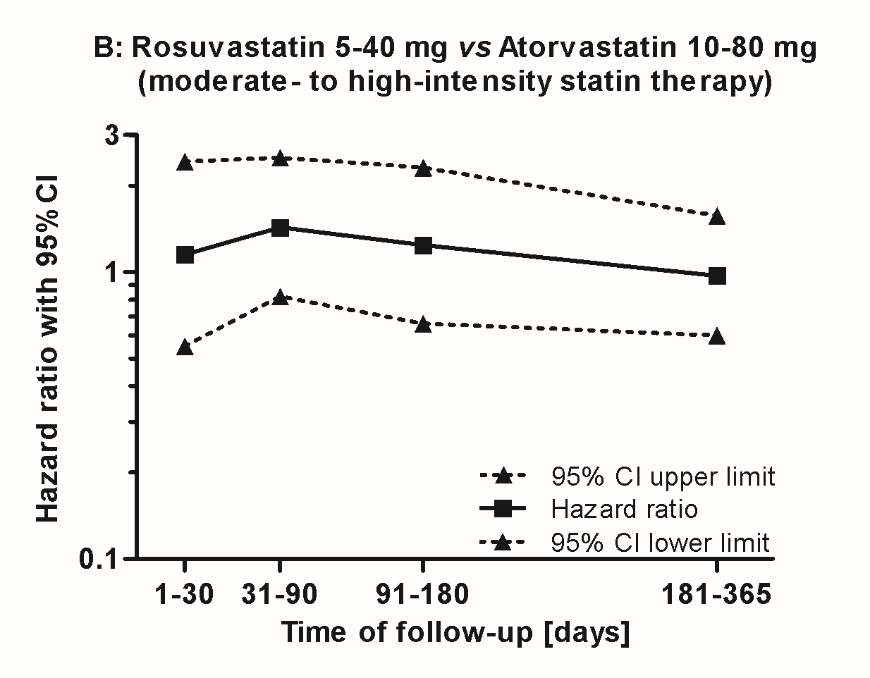

Supplement: Supplementary file 1 — (DOCX 882 kb) [file 11606_2021_6651_MOESM1_ESM.docx]
